# The Cooking and Pneumonia Study (CAPS) in Malawi: A Nested Pilot of Photovoice Participatory Research Methodology

**DOI:** 10.1371/journal.pone.0156500

**Published:** 2016-06-02

**Authors:** Jane Ardrey, Nicola Desmond, Rachel Tolhurst, Kevin Mortimer

**Affiliations:** 1 Liverpool School of Tropical Medicine, Liverpool, United Kingdom; 2 Malawi Liverpool Wellcome Trust, Blantyre, Malawi; Centre for Geographic Medicine Research Coast, KENYA

## Abstract

The Cooking and Pneumonia Study (CAPS) is a village-level randomised controlled trial of an advanced cookstove intervention to prevent pneumonia in children under the age of 5 in rural Malawi (www.capstudy.org). The trial offers a unique opportunity to gain understanding about the social and cultural factors that may facilitate sustained use of improved cookstoves. In January 2015, the use of Photovoice as a participatory research methodology was piloted at the CAPS Chikhwawa site. Photovoice is a photographic technique that allows communities (including women and marginalised groups) to share knowledge about their perspectives and priorities. Four households were given digital cameras and asked to collect images over 24–48 hours and were then interviewed on film about their selection. This resulted in over 400 images and a one hour long film that revealed community concerns and could be thematically analysed. The collection of interview data through film was useful for capturing discussion and was acceptable to participants. Photovoice is a feasible participatory research methodology that can play a valuable role in qualitative studies of improved cookstove adoption in challenging resource poor settings.

## Introduction

Household air pollution (HAP) from cooking using solid fuels in open fires or basic cookstoves causes 4.3 million premature deaths a year [1],[2]. Pneumonia in children, and chronic obstructive pulmonary disease and cardiovascular disease in adults are the main causes of these deaths [2],[3]. Women are particularly vulnerable to adverse health effects due to their prolonged exposure [4]. HAP is intrinsically linked with poverty and the collection and/or purchase of fuel is an additional burden to poor households. The use of solid fuels for cooking depletes forests and the particulates and other partial products of combustion generated (particularly black carbon and carbon dioxide) contribute to climate change.

The use of cleaner burning cookstoves in place of open fires has been proposed as a solution to HAP. Whereas early models were designed simply to use less wood, advanced cookstoves are now being developed that can reduce emissions substantially [5]. The Cooking and Pneumonia Study (CAPS) is the largest trial of an advanced cookstove intervention in the world (www.capstudy.org). The study is underway in Malawi where pneumonia in the under-5s (the primary trial outcome) is a major cause of morbidity and mortality [6]. Additionally in this context there is almost universal use of use solid fuels for cooking [7] and over 50% of the population are defined as poor and 25% as ultra-poor [8].

The adoption of clean cooking technologies by populations targeted in such interventions (particularly over the longer term) is not straightforward. For example, a common occurrence is “stacking”, that is concurrent use of existing cooking methods such as 3-stone fire or basic cookstoves alongside the introduced cookstove. Abandonment of the new technology also often occurs [9]. CAPS offers a unique opportunity to learn more about the social and cultural factors that encourage or militate against adoption of the intervention cookstove with a view to informing future interventions of this type. Qualitative research is an important method for eliciting such factors from the perspective of intervention participants in their social context.

Based on a 2012 systematic review, ‘Who adopts improved cookstoves and fuels’, Lewis and Pattanayak call for a move away from emphasis on individual ‘demand drivers’ and towards a more detailed investigation of the complexity of social systems, which includes gender and household bargaining interactions [10]. In a more recent review in 2014, Rehfuess et al. looked at 57 studies of factors that may encourage or deter uptake of improved cookstoves and identified 31 factors across 7 domains. While the emphasis of this review was the development of more effective planning tools and policy for such interventions, the authors emphasised the need for more good quality qualitative research to investigate the multiplicity of factors that can also be setting specific [11].

As part of a qualitative study, we explored the use of Photovoice in a CAPS control and intervention village in January 2015. Photovoice is a participatory research methodology developed by Wang and Burris in the 1990s and ‘is a process by which people can identify, represent, and enhance their community through a specific photographic technique’ [12]. In seminal works by Wang and Burris, photovoice was shown to be effective in valuing ‘the knowledge put forth by women’ and in making visible the “hidden” context of everyday lives, not just through the images themselves but through the interpretation of the images by the participants [13]. ‘Because virtually anyone can use a camera, photovoice may be particularly powerful not only for women but also for workers, children’, people with limited literacy and marginalised groups in society [12].

Although there is a lack of directly comparative studies, Photovoice has been used in similar environments to rural Malawi where adoption of new practices is seen as key to improving health. For example, in a reflection of the ‘application and utility of photovoice for understanding water, sanitation and hygiene (WASH) behaviours’, in a study in rural Kenya, the authors conclude that the methodology was particularly useful to explore the ‘complexities of water-related behaviours in the community that other research methods such as surveys and interviews may not fully capture’. The efficacy of the methodology in understanding behaviour is emphasised and in particular, its ability to capture the ‘social dimension’ of everyday practices, encouraging critical reflection and providing a mechanism of communication between the participants and researchers [14]. These benefits are also relevant to studies of cookstove adoption.

The theoretical basis of this pilot work is the anthropological concept of culture as a complex interaction between individuals and groups that needs to be examined as processes are enacted [15]. The identification of social phenomena in naturalistic settings through the use of qualitative and participatory methodologies is key to revealing the perspectives and priorities of the research subjects in cookstove intervention trials. In addition, these methodologies can be used to gain further understanding about the gendered and household dynamics that contribute to shaping the adoption of improved cookstoves. Wallerstein and Duran identify community-based participatory research ‘as a transformative research paradigm that bridges the gap between science and practice through community engagement and social action to increase health equity’ [16].

Photovoice methodology is underpinned by three theoretical foundations. First Freire’s idea of critical consciousness arising through dialogue with and education of the “oppressed” [17]. Second and in response to criticism of Freire for ignoring the dominant power of men in societies, Photovoice incorporates feminist theory with the aim of facilitating both the involvement of women and their empowerment, from the starting point of giving them the means to represent their own perspectives and experiences beyond dominant narratives. The third foundation is community based documentary photography ‘as a way of thinking about how ordinary people could appropriate the camera for social change’ [18].

The use of visualisation within participatory research through photos, maps, drawing or drama has been shown to have many benefits, particularly when working with marginalised groups. Whereas interviews can be seen as interviewer directed, these techniques encourage participants and researchers to work together collectively, with the potential to shift power relations between researchers and researched. ‘The visualized product acts as a focus and anchor for discussions’ and ‘offers a powerful strategy for working with those whose voices are rarely heard, as well as for bringing about critical awareness and self-confidence among participants in the process’ [19].

In this paper we describe our experience of the photovoice methodology in rural Malawi, share lessons learnt and make recommendations for the use of this methodology in future cookstove intervention studies.

## Materials and Methods

The aims of this pilot study were to assess the feasibility and potential of Photovoice for qualitative research on understanding cookstove adoption under CAPS and to identify any logistical, ethical and other issues associated with the methodology in this context.

**Fig 1:**
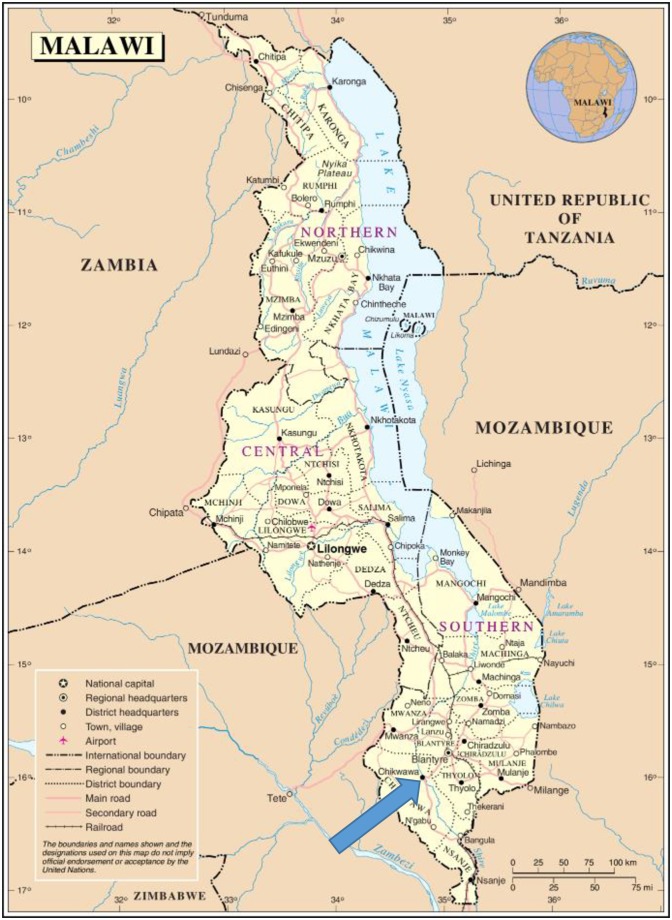
Map of Malawi. Chikhwawa in shown highlighted, south of Blantyre, the commercial capital of Malawi (based on United Nations Map no.3658).

CAPS has two study sites: Chilumba in the far north of Malawi, and Chikhwawa, one hour south of Blantyre (see Fig 1). The selected site for this study was Chikhwawa. This is the base for the Malawi-Liverpool-Wellcome (MLW) Chikhwawa Child Survival site and is a low lying area with high a disease burden (including malaria), poverty and food insecurity. As an established field site with an experienced field team, the MLW base provided the resources necessary for this pilot study including field work support and assistance with photo processing. The CAPS participants are part of an established “researched community” and enrolled in an ongoing trial and as such had shown their willingness to be involved in research.

The CAPS field team at Chikhwawa were initially provided with a guidance document introducing them to the Photovoice concept and outlining the technique. The first author (JA) liaised with the Senior Fieldworker to arrange recruitment of four households enrolled in the CAPS study, two in a control village and two in an intervention village, and requested that the participants included a female-headed household.

Selection of villages was done in a pragmatic way with ease of access to the CAPS field site an important factor, especially as the pilot study was conducted in the rainy season and coincided with major flooding in the area (http://www.bbc.com/news/world-africa-30805498). Similarly, no attempt was made to recruit a representative sample of the CAPS population. However a variety of participants were purposively recruited as follows: a female head of household; a younger married couple; an older married couple; a male (married) petty trader. Therefore within the context of this pilot study, the principle of maximum variation was applied. This resulted in a sample size of 4 households and 6 participants.

The involvement of CAPS fieldworkers was crucial to this pilot study, both in the selection of participants and for facilitating the process. Since their knowledge of participatory research methods was limited, two information sheets were produced by the researcher, one for fieldworkers and one for participants. In addition, a topic guide was produced and JA emphasised the importance of allowing the participants to discuss their own concerns and of not asking leading questions. A local Malawian filmmaker was contracted to train participants in camera use and conduct and film the interviews.

The pilot study was conducted between the 14^th^ and the 17^th^ of January 2015; training and discussion with the participants took place outside the participant homes as is usual in this context. The pilot study team included the CAPS Senior Fieldworker and three CAPS Fieldworkers who are all Malawians and experienced in their roles. The local filmmaker had previously been employed by the BBC to collect footage for a film about CAPS (http://www.bbc.co.uk/news/health-30449431) and clearly had the ability to communicate effectively with the study participants. He was accompanied by an assistant whose input was purely technical. JA is European and was responsible for study design and data analysis and accompanied the other team members in the field to oversee the process. She is an experienced programme manager, familiar with the CAPS context.

In stage 1, all participants were visited at their homes and trained in camera use by the film maker (this process took approximately 30 minutes per household). They were asked to take any images relating to food and drink over a period of 24–48 hours. The subject matter was guided by the requirement of the funder but was not restricted further in any way. It was made clear to the participants that the choice of images to capture was entirely their own, with the caveat that at the discussion stage we hoped that they would be able to link their image with food and drink either specifically or more generally.

At stage 2, the cameras were collected from each household, participants were asked about any problems they had encountered and an appointment was made to come back and discuss the photographs. The memory cards from the cameras were taken to Blantyre, the nearest city about one hours drive away, for processing. Once collected, the prints were checked for quality and content: that is, that the printed photographs were the same as the digital images.

For stage 3, appointments were made to meet with the four households at their homes and to interview the six participants discussing their images. The pilot study team met beforehand to go through the process and the researcher checked that all were familiar with content of the information sheets and topic guide. To expose the fieldworkers to unfamiliar qualitative research techniques and to free up the filmmaker to concentrate on collection of footage, each of the fieldworkers was assigned to carry out an interview or interviews at one of the participant households.

Each of the interviews was filmed and all participants were asked to sign to give permission for use of the footage and images before the process began (using locally approved forms). Personal microphones were used to record sound and a film and still camera were used concurrently to collect footage.

In recognition of the time and effort of the participants, they were offered a choice of any of the printed images at the end of the discussions. This was not indicated to them beforehand in case this influenced their selection of images. The children in each household were given very simple, low cost toys to keep them occupied during the discussion.

Feedback from the participants and CAPS fieldworkers was sought by JA at two stages. The CAPS Senior Fieldworkers visited the participants at their homes approximately one week after the pilot work was completed and asked for any comments about what went well and what did not. In addition, JA solicited comments from the fieldworkers immediately after the pilot study and also asked the team to report back any additional positive or negative opinions via e-mail.

In August 2015, JA returned to the Chikhwawa field office to disseminate the pilot study results through meeting with the field workers and the participants. She used a scientific poster describing the pilot study as a reference point and also showed the short film. Both activities took place outside the Chikhwawa base in a gazebo used for meetings. The participants were transported to and from the site using the CAPS vehicle and were each given a study t-shirt in recognition of time away from their usual activities. The younger couple were not able to attend as they were attending a funeral.

The filmmaker provided a copy of the full footage and this was transcribed and translated by a Malawian researcher based in the UK. One of the CAPS fieldworkers then checked the complete transcript for accuracy of transcription and translation. The final full translated transcript was analysed by the author using a Framework approach to identify themes inductively, with a view to assessing the value of Photovoice as a methodology in this context [20]. The researcher familiarised herself with the data set and also studied the full (untranslated) footage. Inductive codes were assigned to each section of transcribed data and these were charted in an excel spreadsheet.

The Malawian filmmaker was tasked with producing an excerpt film from the footage for presentation at the Wellcome Trust sixth annual International Engagement Workshop “Global Food Matters: an appetite for research” which took place in Botswana February 2015 [21]. Due to time pressures, he made the decision about what to include. In the film, each participant briefly introduces themselves followed by discussion of the images which is interspersed with the images themselves.

The qualitative research theme of CAPS was approved by the College of Medicine Research Ethics Committee (COMREC) in Malawi and LSTM Research Ethics Committee (REC) in the UK. Informed and voluntary written consent was obtained from all study participants. The individuals in this manuscript have given written informed consent (as outlined in PLOS consent form) to publish these case details.

## Results

**Fig 2:**
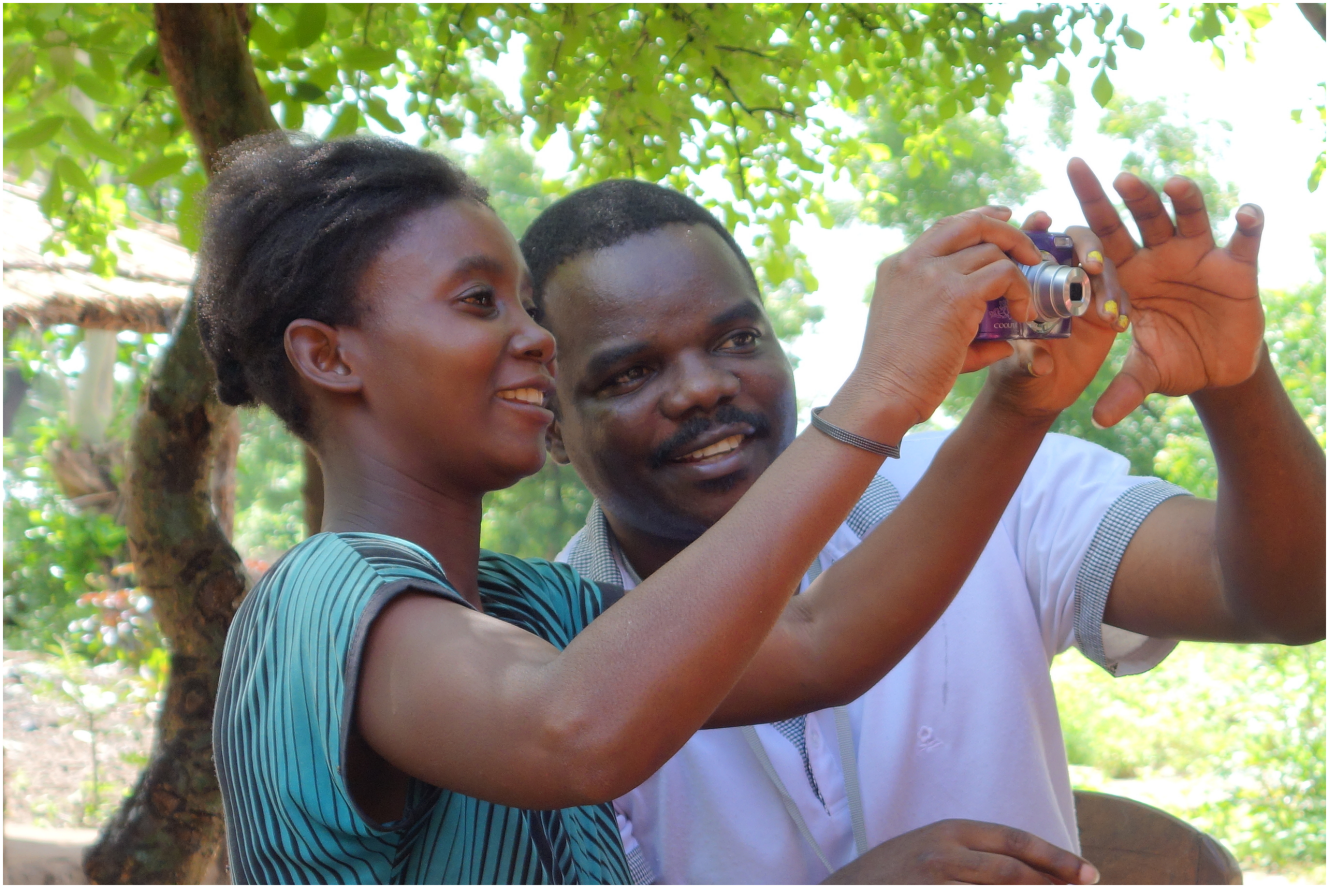
Camera Training 1. The younger female participant practising turning the camera on and off, assisted by the local filmmaker.

**Fig 3:**
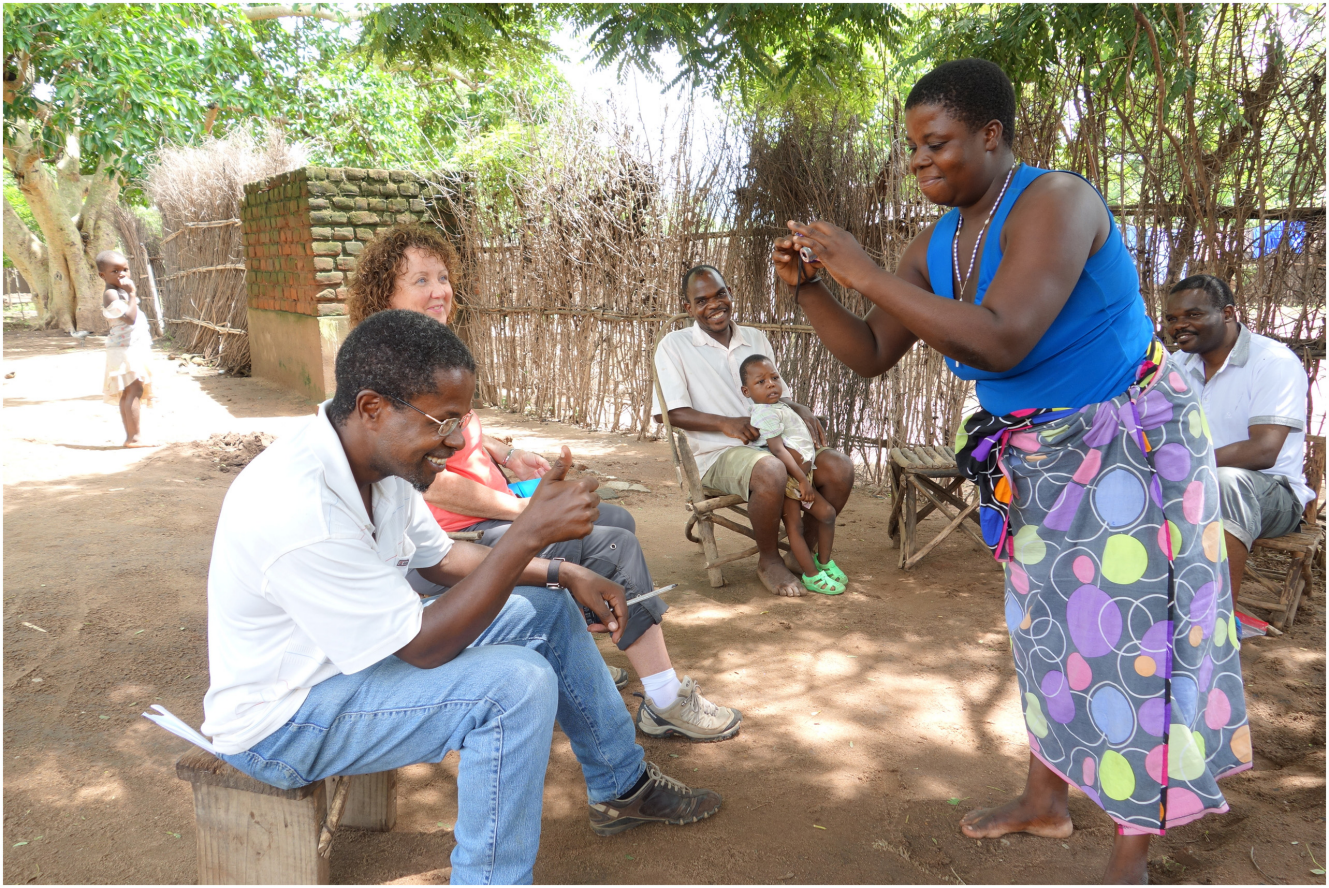
Camera Training 2. The older female participant capturing a test shot of the CAPS Senior Fieldworker.

From the initial stage of training participants in camera use, it was clear that there was considerable enthusiasm to collect images. In particular, two individuals said that they had already scoped what images they would take and in general all were keen to get started. Although only one of the participants had previously used a camera, the training was straightforward and both the technology and task seemed acceptable to the six individuals. See Figs 2 and 3 for images of participant camera familiarisation.

When the cameras were collected, the participants were asked about any issues that they had encountered. Two participants reported that when taking pictures in public places they had been asked what they were doing and some people had refused to have their picture taken. Their explanations led to further discussion including queries about whether the camera was a gift for participation and if not, why not, that is, should participation be financially rewarded? In both cases, these encounters did not cause concern to the participants and they had been happy to explain that they were content to borrow the camera and take part in the research.

When the images were processed, it was noted that although there were several pictures showing cooking on open fires and fuel collection, there was only one showing the intervention cookstove. It seemed therefore that the presence of the CAPS fieldworkers did not appear to have influenced the image collection; that is, there was no indication that participants had set out to show their compliance with the intervention. The initial plan for the filmmaker to carry out the interviews was therefore changed and it was decided that the fieldworkers could have an active role in this process. Example images are available in S1 File.

At the interview stage, the first participant to be interviewed was the female head of household who had taken the largest number of images (157) and it took a while to sort these (on flipchart paper on a mat on the ground). There was also unfamiliarity with the process on both sides and in particular, the interviewer had concerns about the open-ended nature of the discussion due to familiarity with quantitative data collection through the use of questionnaires in CAPS. The researcher therefore needed to re-emphasise that the aim was to encourage the participant to talk freely about why a particular image or set of images was collected. However, once these initial issues were dealt with, the participant talked with confidence and at length; the whole process took 1 hour, 40 minutes. The researcher was able to observe this process and to ask other fieldworkers present for translation when required without interrupting the flow. In the subsequent discussions there was again some initial hesitancy on both sides but participants were clearly keen to explain their choice of images and to share their experience and knowledge through this medium.

In all cases, decisions about how to sort images, whether to talk about groups or individual images or to omit discussion of certain images was made by the participants. For example, portrait shots of family members with no food and drink context were put to one side. The interviewers facilitated in-depth discussion by encouraging the participants to group their images together and prompting discussion of all images they had selected. That is, the interviewers simply encouraged participants to explain the “story” behind their images

The process did not attract a great deal of attention from the village population although during the first discussion neighbours gathered around and the fieldworkers did at times need to ask individuals (particularly children) to move out of shot. The time spent on discussion was aligned with the number of images taken; for example to discuss the 48 images selected by the young couple took 50 minutes.

**Fig 4:**
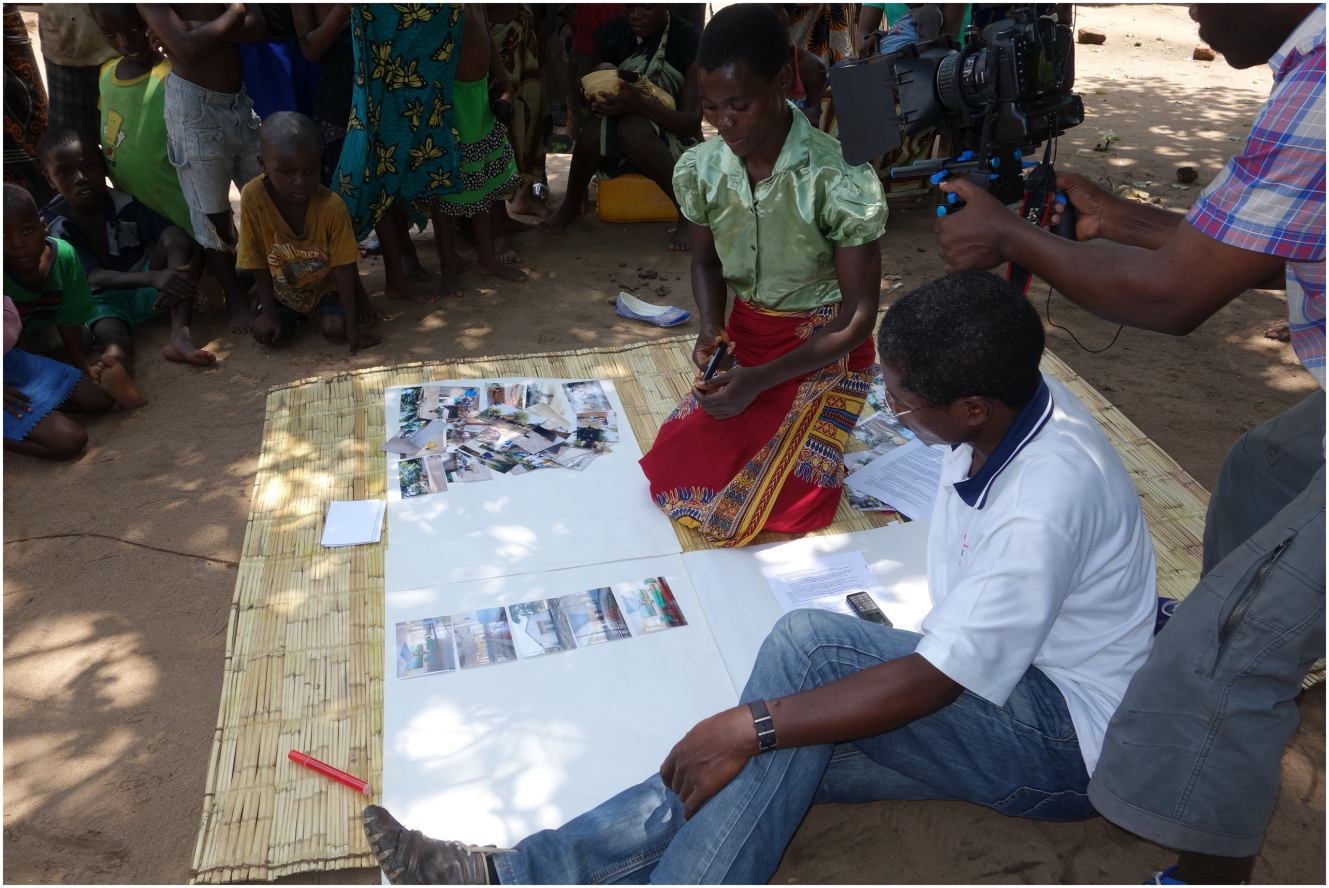
Interviewing Process. The female head of household explaining her image collection.

Activities such as camera training and interviewing took place outside participant’s homes (see Fig 4) and this would not have been possible if it rained. This was a concern as the weather in Chikhwawa at this time of the year can be very wet and in January 2015 there was major flooding in the area. In rural villages in Malawi social activities take place in the open and moving these activities indoors may have been problematic and would certainly have changed the dynamic.

The short film produced from the footage is simple in style although professionally produced. By combining film of the participants with their images it provides a mechanism for the participants to share their life experience directly and effectively with a wide audience, (see link here). The full film was shown at the Botswana workshop and shorter excerpts have been included in subsequent scientific presentations made by the researcher.

**Fig 5:**
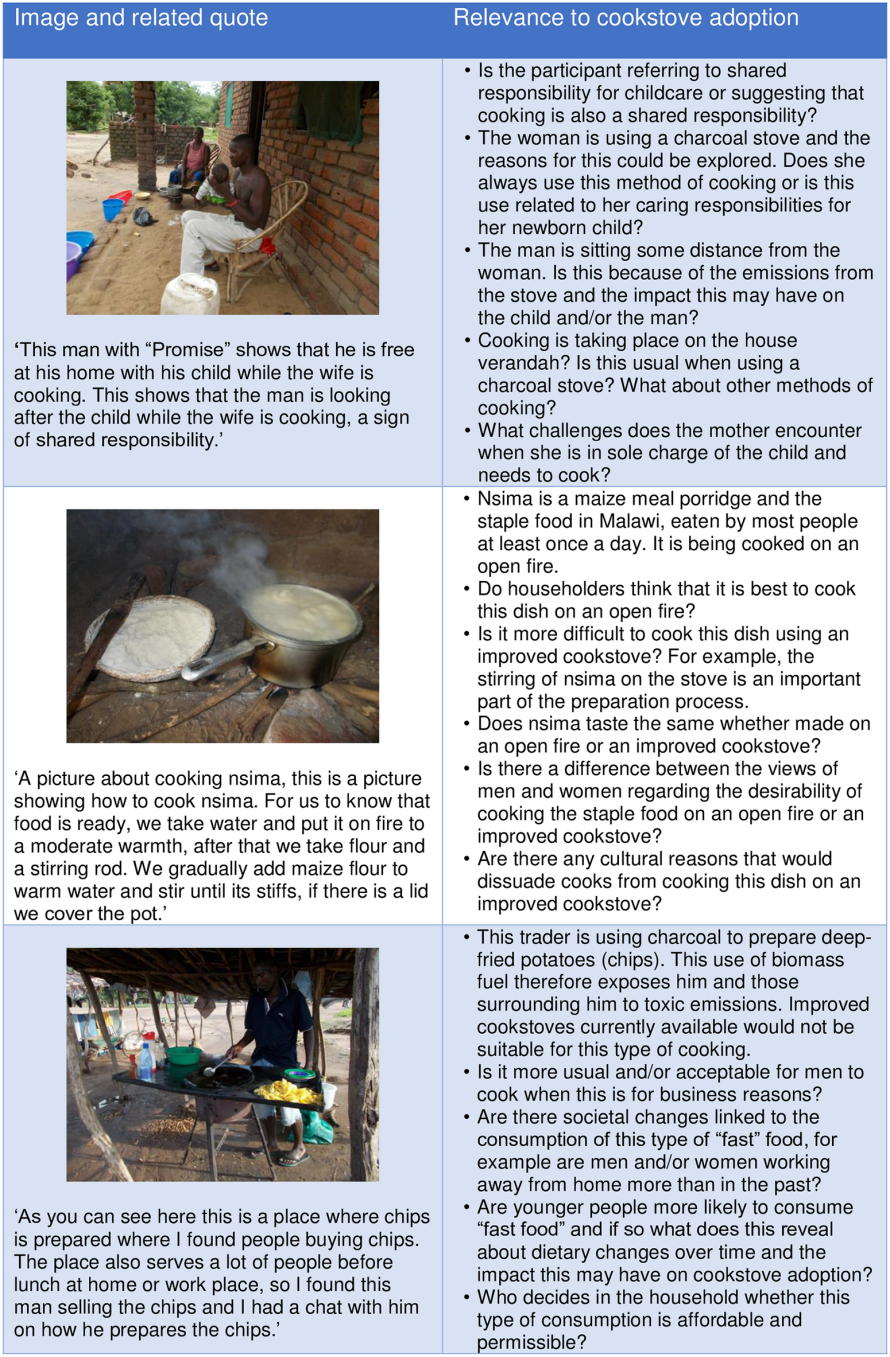
Linking pictures and text to cookstove adoption issues. Example quotes and associated images are displayed with suggestions for further cookstove adoption research questions and areas that could be explored through qualitative research.

The translated transcript is a rich source of data and themes such as sharing of food diet, food purchase and trade, could be identified. In addition it became clear that these themes could be conceptually related with sociocultural, economic and health domains. See Table 1 for a full list of themes with illustrative quotes and relevant domains. Fig 5 shows how images and themes can be related to cookstove adoption issues.

**Table 1:** Full list of themes arising from data analysis. Themes are shown with illustrative quotes and associated domains.

*Theme*	*Domain*	*Quote*
**Cooking Fuel**	Economic Health Sociocultural	‘There, that picture shows the charcoal stove that is used when we have no firewood for cooking, we set fire using charcoal in the stove as an alternative.’
**Diet**	Economic Health Sociocultural	‘Sometimes when one is hungry we just eat them (bananas) directly from the garden as we can see the fruits hung up…we cut bananas and make breakfast out of them, locally called makata and sometimes we store them until they are ripe.’
**Food availability**	Economic Health	‘Food is never scarce this time due to rains. The fields have okrah which we can cook for relish.’
**Food Hygiene**	Economic Health	‘You can see this is unprotected salad we normally consume when we want to buy chips…flies can contaminate food and cause diseases.’
**Food preparation**	Sociocultural	‘We detach them (Msangu tree leaves) and put them in a winnower then set fire and put some water until they boil. While the water is boiling, we pound some groundnuts as I am doing here.’
**Food purchase and trade**	Economic Sociocultural	‘This is the first picture, some of the people around here are fishermen, they usually walk around the village with the fish we buy from them using the money we get from piece-works. Secondly, these fishermen catch and sell the fish in order to find money for their day to day lives.’
**Growing food**	Economic Sociocultural	‘This is our new type of farming because most of the times the fields where we farm here in Mlinga village are on a distance so we thought of a small garden around our home to grow vegetables for food.’
**Sharing of food**	Sociocultural	‘Most of the times the people who are around are kids. We sometimes share food with them when they are coming from school while their parents are at farms.’
**Waste disposal**	Economic Health	‘This is tomato and a bin. Instead of disposing in a bin, they have carelessly disposed it, which is unclean as seen from this picture, an act that is not acceptable.’
**Water safety**	Health	‘We used to collect water from the Shire River which was an unsafe practice that was causing a lot of diseases in our family. After they installed tap water, I can say things have improved. We have been using safe water for cooking and washing.’

The post pilot discussion with the CAPS field team (not just the pilot study team members) in August 2015, was lively and prompted discussion of the difficulty of soliciting information about why participants might not use the intervention cookstoves. It was pointed out that it is important to look at the possible benefits of the intervention as well as “difficulties”. The gendered aspect of cookstove use and household decision was also highlighted and there was general agreement that an understanding of gender in the context of rural Malawi would be important for further qualitative research within CAPS. An important comment (by a male staff member) was that men are considered to be the “facilitators” in Malawian society; that is the group that makes things happen.

The feedback from the pilot participants shortly after the study completion was that it would have been useful to have more time and resources, to gather more images and expand the scope of enquiry. When JA returned and showed the film and displayed the scientific poster, the response was also positive. The group (which excluded the younger couple) reported that they had enjoyed the process and hoped to be involved further if possible. They appreciated being kept up to date with what had happened with their images and the film and asked if they could each have an (A4) copy of the poster; this was provided.

## Discussion

This pilot work was completed successfully, demonstrating that the methodology used is feasible in this context. With the assistance of an experienced field team, input from a skilled filmmaker and with oversight of JA the different stages of the process were accomplished within the set time frame and the outputs were rich data that could be thematically analysed and an engaging short film.

The potential of participatory research methodologies in rural contexts such as Chikhwawa is well established and the use of visualisation techniques is rooted in Participatory Rural Appraisal (PRA). Writing in 1992, Chambers reported that ‘evidence to date shows high validity and reliability in information shared by rural people through PRA’ [22]. This pilot study adds to the evidence base for expanding the use of Photovoice as part of a participatory methodology “toolbox” in rural environments in sub-Saharan Africa and similar contexts.

This pilot study found a number of benefits to the use of Photovoice methodology in the setting of a large-scale cookstove intervention trial. Both fieldworkers and participants welcomed the opportunity to participate in a “new” type of research. The images collected and themes arising showed that participants looked beyond what may have been seen as CAPS priorities, such as smoke emissions and fuel use, and were able to express their own concerns and priorities. When looking at the complex cultural and societal factors that are “naturalised” in everyday activities such as cooking, the use of techniques that break down the barrier between researchers and participants and create a shared “text” for analysis between people with different educational levels is a valuable asset.

The images and discussion data reveal that issues around food and drink involve a complex interaction of economic, health and sociocultural factors. Reviews of qualitative research into cookstove adoption indicate that identifying multi-factorial drivers of large-scale sustained clean cooking technologies is key but difficult to achieve [10, 11, 23]. This pilot study illustrates how Photovoice can be used to elicit themes that can then be explored in more detail through interviews, focus groups and other qualitative research methods. Wang suggests the use of SHOWeD methodology to examine in more detail issues raised through image collection. That is by asking: ‘What do you **S**ee here; What’s really **H**appening here? How does this relate to **O**ur lives? **W**hy does this problem, concern, or strength exist? What can we **D**o about it?’ [24]. This technique was not used in the pilot study but will be incorporated into further planned research on cookstove adoption using Photovoice.

As the SHOWeD methodology is designed for needs assessment in the context of participatory research designed to empower participants, it would be necessary to modify this methodology for examining factors associated with cookstove adoption. An example of a modified (essentially truncated) use of the acronym is described by Freedman in a study using photovoice to identify key socio-environmental factors impacting on the health and well-being of a public housing community [25].

Logistical challenges mainly centred on the village outdoor life. All activities took place outdoors and the heavy rains encountered in this area in November to January could have been problematic. An alternative venue, a community hall, was considered but fortunately did not need to be used. Moving the participants away from their familiar setting would in this case have introduced a more formal atmosphere at odds with the Photovoice methodology. The processing of the images collected entailed a fieldworker travelling to the nearest town one hour away and waiting for processing before returning. Depending on when initial training and interviews took place, participants had between 24 and 48 hours to collect images but even in this short time captured many images; by household the figures were 48, 80, 136 and 157. In a larger scale study it would therefore be cost-effective and practically simpler to restrict the number of images that could be collected.

The ethical issues arising from the use of Photovoice in this context are varied and complex. Most straightforwardly, the safety and security of the participants was carefully considered before progressing. As illustrated by the fact that only one participant had previously used a camera, ownership of such technology is not normal in this context and there was a concern that theft or attempted theft may have exposed participants to harm. After discussion within the pilot study team, the consensus was that theft was not likely in the village context where it would be impossible to use the camera or sell it without attracting considerable attention. The low level of theft of the CAPS cookstoves was also an important consideration.

In this pilot study, the local expertise of the pilot study team (the CAPs field workers and the local filmmaker) was key to assessing risk and also any unintended consequences of introducing cameras and soliciting the collection of images. The researcher initiated a discussion of anticipated issues before the study started and any concerns were carefully considered. In some communities, photography may be associated with witchcraft and distributing cameras may disrupt community dynamics. It is therefore important for these type of issues to be explored before any use of Photovoice in a community [26].

The collection of images in public is also an activity that could expose participants to risk. Through the participant information sheet and during training in camera use, participants were advised to take care when doing this and to not insist on taking images if they encountered any resistance. Describing lessons learnt from a large-scale Photovoice project in the United States, the authors suggest that participants should give out written information to those they photograph, get signed permission and also offer them a print of the image [18]. The low levels of literacy in the Chikhwawa community and the inability to get images printed locally made both suggestions infeasible. However, in any larger scale study involving Photovoice in this context, further consideration will need to be given to this matter. For example, role play could be used in training to prepare participants for any such issues in case they arise.

In this case we did not aim for the data collection to lead to any changes in participant communities, and we endeavoured to “manage expectations” of the outcomes of the study by explaining from the outset how the images and data would be used. JA showed the film and a scientific poster to the participants in August 2015 to illustrate these outcomes and the “pilot” nature of the study was reiterated at every stage. In a larger scale participatory study using Photovoice the dissemination of results should be given careful consideration, for example by sharing the results with bodies such as the Malawi Ministry of Health. In addition presentation of the images in a public forum could be included.

With regard to empowerment of the participants, this was not formally assessed as part of this pilot study but there was relevant feedback from the participants about an increased sense of expertise and associated sense of pride. Specifically, the female head of household reported that through her experience of camera use she had been able to respond to a call for participants in another research project conducted by Malawi local government. The petty trader also made the suggestion that the six participants could be considered ‘local experts’ in any further use of this technique in the CAPS trial and others present agreed that they would also like to be involved.

As Dockery outlines, pure emancipatory, empowering participatory research is seldom achievable, ‘what is important is that we look purposefully for entry points where participation can be facilitated and attempt to push the boundaries further along the continuum towards the ideal’ [27]. The distinction made by Guijt and Kaul Shah between participation as a ‘means’ or an ‘end’ is also helpful in this context and according to their definition, it is valid to take an ‘instrumental approach’ to participatory research as opposed to an ‘empowerment approach’ [28]. That is, although in some cases the primary aim of participatory research is empowerment, these methodologies can also provide the means to investigate issues such cookstove adoption, but may have secondary outcomes related to empowerment. In incorporating Photovoice into qualitative research in this field of enquiry it will be necessary to take a pragmatic approach. However, the process may still be conducted in ways that disrupts the conventional power balance in research. In addition, the generation of trustworthy data relies on an appreciation of power relations, such as gender, within the research context.

Gender is a key dimension to cookstove adoption studies and also to the use of participatory methodologies. The link between gender and cooking can straightforwardly be explained as follows: women and girls spend a disproportionate amount of their time collecting fuel and cooking over smoky fires, which has a detrimental impact on their heath, but men largely decide whether or not to buy an improved cookstove [29]. However, factors such as whether women have *de facto* decision making power about use of cookstoves, also need to be considered. The complexities of household decision making and bargaining are nuanced and difficult to explore [30]. Participatory research methodologies, including Photovoice are useful in this context as such techniques provide a way to explore “hidden” roles and processes embedded within social norms. It is important, however, to ensure that male and female voices are heard and that the researcher retains an awareness of gender relations and their often hidden influence on daily activities and decisions.

To our knowledge this study is the first to use Photovoice methodology in the context of a cookstove intervention trial. In addition, we were not able to find any other examples of this type of participatory research in rural Malawi. In a review of Photovoice literature in health published in 2009, Catalani and Minkler, identified 37 unduplicated articles referring to use of this methodology. The majority of these studies took place in the United States but four studies were carried out either partially or completely in communities in Africa. Specifically, working with: paraplegic Cameroonians to enhance understanding of the burden of disease; Ugandan nurses to gain more understanding of their needs and assets with a view to improving HIV patient care; Batswana teenagers to engage this group in HIV prevention and education; South African gay and lesbian adults to discover more about their assets and needs and to counter discrimination and empower individuals [24].

After the completion of this pilot study, a report was published describing the use of participant photography in research into the behavioural drivers of the use of cookstoves in Kibera, a large informal settlement in Nairobi Kenya. The authors’ theoretical approach is informed by behavioural economics and anthropology and incorporates the research method of ‘photo-elicitation’, defined here as a ‘cultural probe’. The authors report that although they did not examine in detail the effect of using the cultural probe, they did note a difference in interviews when photographs were not used due to technical issues. Specifically, they observed that the dynamic shifted when photo-elicitation was used: it was much easier to establish rapport and the process encouraged the interviewee to have a more assertive role. They conclude that ‘the photographs served as a way to shift the physical focus away from the respondent, relieving some of the pressure on them to “perform” or say the right thing’ [31].

This may be reflected in the results of this pilot Photovoice study in that participants did not emphasise issues such as emissions from cooking or use of the intervention cookstove, suggesting that the method may have helped to overcome any limitations on their responses that might have been created by straightforward interviewing by CAPS fieldworkers. Instead a variety of concerns was raised and the participants also used the process to share the “taken for granted” detail of their everyday cooking and food preparation practices.

In their review of Photovoice, Catalani and Minkler used the tool formulated by Viswanathan et al. in 2004, to rate the level of community participation in each study [24]. The authors concluded that shorter participatory studies tend to be of lower quality and omitted key parts of the Photovoice methodology such as ‘dialoguing with other project participants about the photographs and their meanings’. Only higher quality more participatory studies were likely to achieve the three goals originally identified by Wang and Burris. That is: ‘to enable people to record and reflect their community’s strengths and concerns, to promote critical dialogue and knowledge about important issues through large and small group discussion of photographs, and to reach policymakers’[12].

This pilot study was clearly a truncated version of Photovoice methodology and was limited in time and size. Participants had a short time to collect images and it was not possible to facilitate group discussion of images. In a larger scale study it would be possible to build a longer term, closer relationship with the participants and hold group sessions where images can be critically analysed by the group

As previously stated, empowerment of the participants was neither a specific goal of the pilot study or measured systematically. It must however be acknowledged that using photovoice methodology merely to extract data is a limited application of the methodology and presents ethical challenges. With regard to future research on cookstove use, there is a possibility that participants will become more self-reflexive and have their consciousness raised by the process but not have the means to effect change. It is incumbent on researchers to make every effort to “move along the participatory continuum”, by using every available opportunity to acknowledge the significant input of the participants and sharing the outputs from the research process as widely and strategically as possible. In this pilot study, dissemination of the results within the community is an important and continuing process. The participants may also benefit from being part of a Chikhwawa field site research community with opportunities to be involved in other research studies and/or take on MLW community liaison roles.

It is a strength of this pilot work but also a limitation on its wider applicability that participants were recruited from an existing cohort of CAPS enrolees and research was carried out in the context of a large scale trail in an established research community. This undoubtedly contributed to the success of the pilot. By agreeing to participate in CAPS, those recruited had already indicated their willingness to be involved in research and had an established relationship with the field team. However, conducting research into cookstove adoption outside of this type of context may be more challenging so wider application of the findings of this study may be limited in this respect.

It was a strength of the pilot study that we allowed participants to choose their own issues to explore (within the context of food and drink) but when looking at cookstove adoption it will be necessary to narrow the focus of enquiry, resulting in more direction by the researcher. However, there are clear indications that involvement in this type of research in this context encourages participants to raise issues that are important to them as individuals as well as to the community. They are experts on their own lives and are provided with a mechanism for sharing their knowledge.

The use of film in this pilot study was prompted by the funding mechanism and while this may be seen as an optional (and potentially expensive) extra, on reflection it added an important element to the research. The full film was shown at the Botswana workshop and it has been possible to include a one minute excerpt in several presentations. On each occasion this has prompted a lot of interest and led to further discussion. In the context of scientific conferences and meetings, it is unusual but powerful to have a research participant speaking on film about issues that matter to them personally; to be “in the room”. In addition there is clearly an opportunity to use such footage to share the lived experiences of marginalised groups and advocate for change.

## Conclusions

We conclude that Photovoice is a flexible participatory methodology and can be adapted pragmatically to explore factors that impact on the adoption of improved cookstoves; the value of this approach is strengthened by aiming for the highest level of community participation possible [24].

We plan to incorporate Photovoice into the qualitative research within CAPS and will use this pilot study to inform our research design. We will carefully consider our participant selection with an emphasis on the gendered dimension of both cookstove adoption and participatory methodologies, recognising that our enquiry must include men, as well as women. The time for collection images will be expanded but we will restrict the number of images that should be collected for cost and logistical reasons. To allow participants to concentrate on the defined subject area we will agree to print a set number of family images for the participants to keep. There will be an additional focus group stage to the process so that groups of participants can meet to discuss images and prioritise issues communally. It is unlikely that our budget will allow for filming of all activities but we will look for opportunities to gather film footage that can be used for community engagement and advocacy.

We hope that other researchers in the improved cookstove field will build on our experience and expand the use of this methodology. The challenge of stove “stacking” and the difficulty of establishing sustained adoption of clean cooking technologies are well recognised. Exploring the “taken for granted” and “hidden nature” of the everyday activity of cooking is difficult, but this pilot study indicates that Photovoice can be engaging for participants and generates rich data for researchers and as such can be an important tool in qualitative research of cookstove adoption.

## Supporting Information

S1 File Example images collected by participants. (PDF)

S2 File Transcript data. (PDF)
