# Supplementary figures and images for: The Cooking and Pneumonia Study (CAPS) in Malawi: A Nested Pilot of Photovoice Participatory Research Methodology
Source: PLoS One. 2016 Jun 2;11(6):e0156500. doi: 10.1371/journal.pone.0156500 (PMC4890783; doi:10.1371/journal.pone.0156500)

S1 – Example images

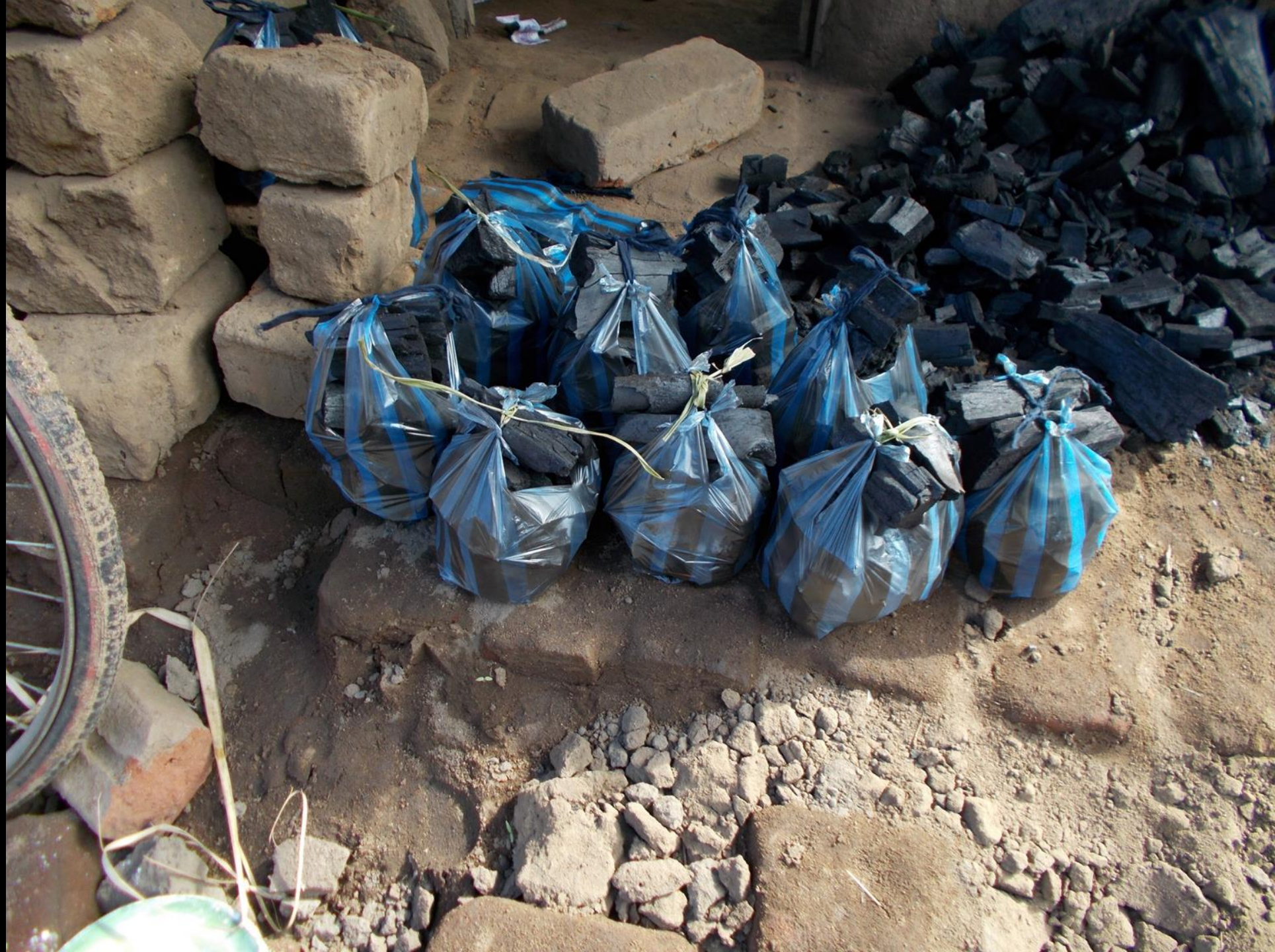

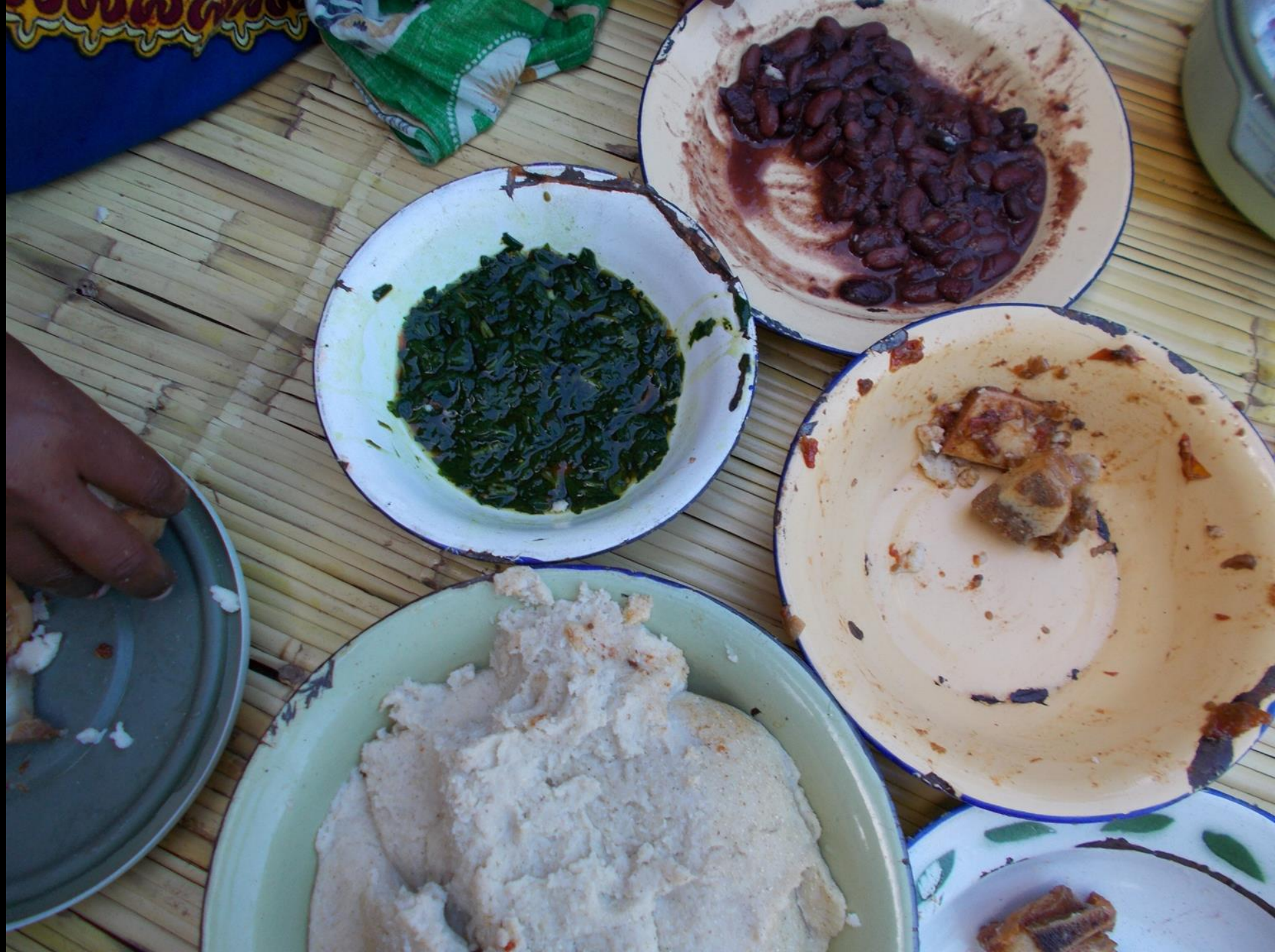

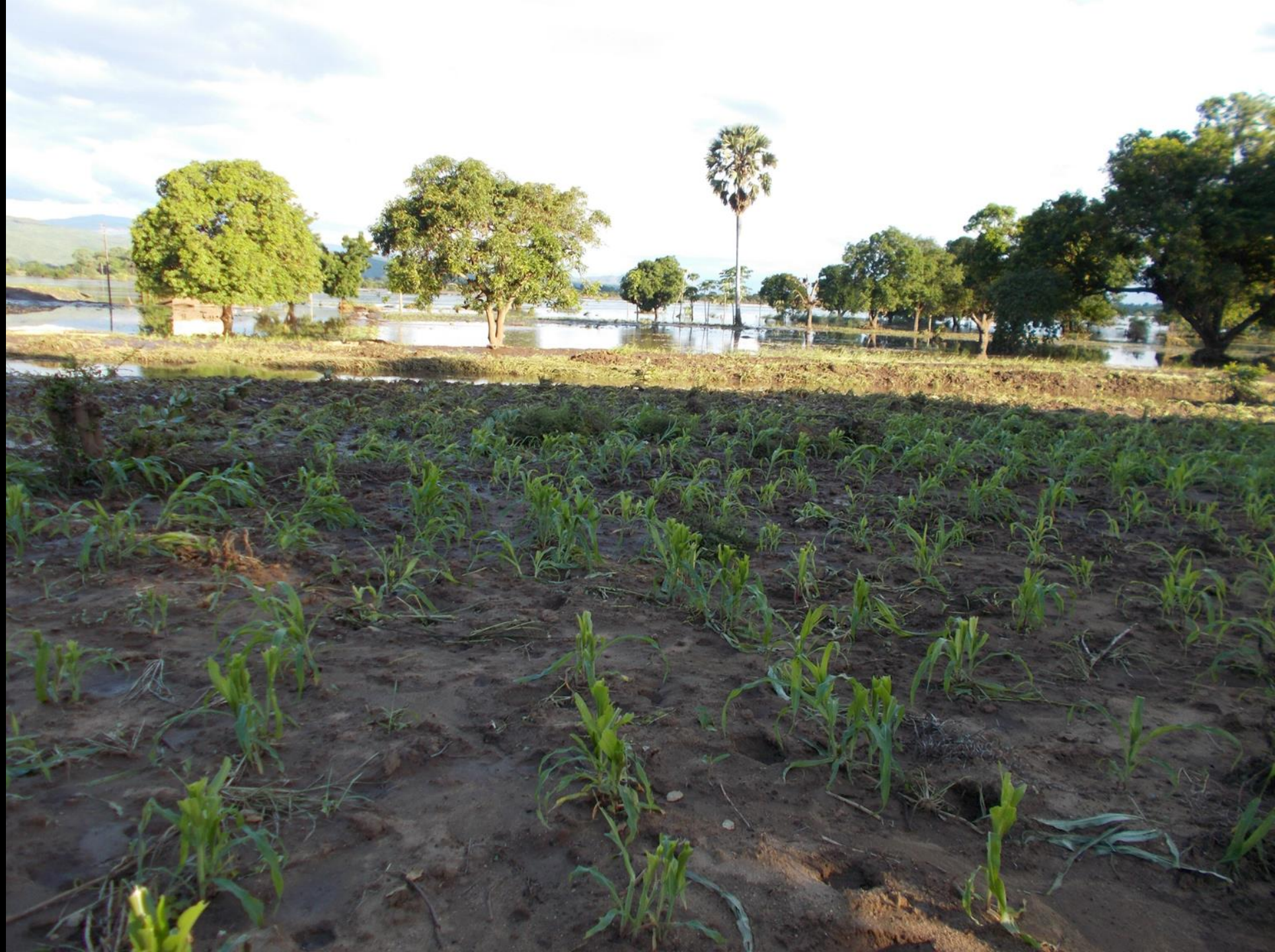

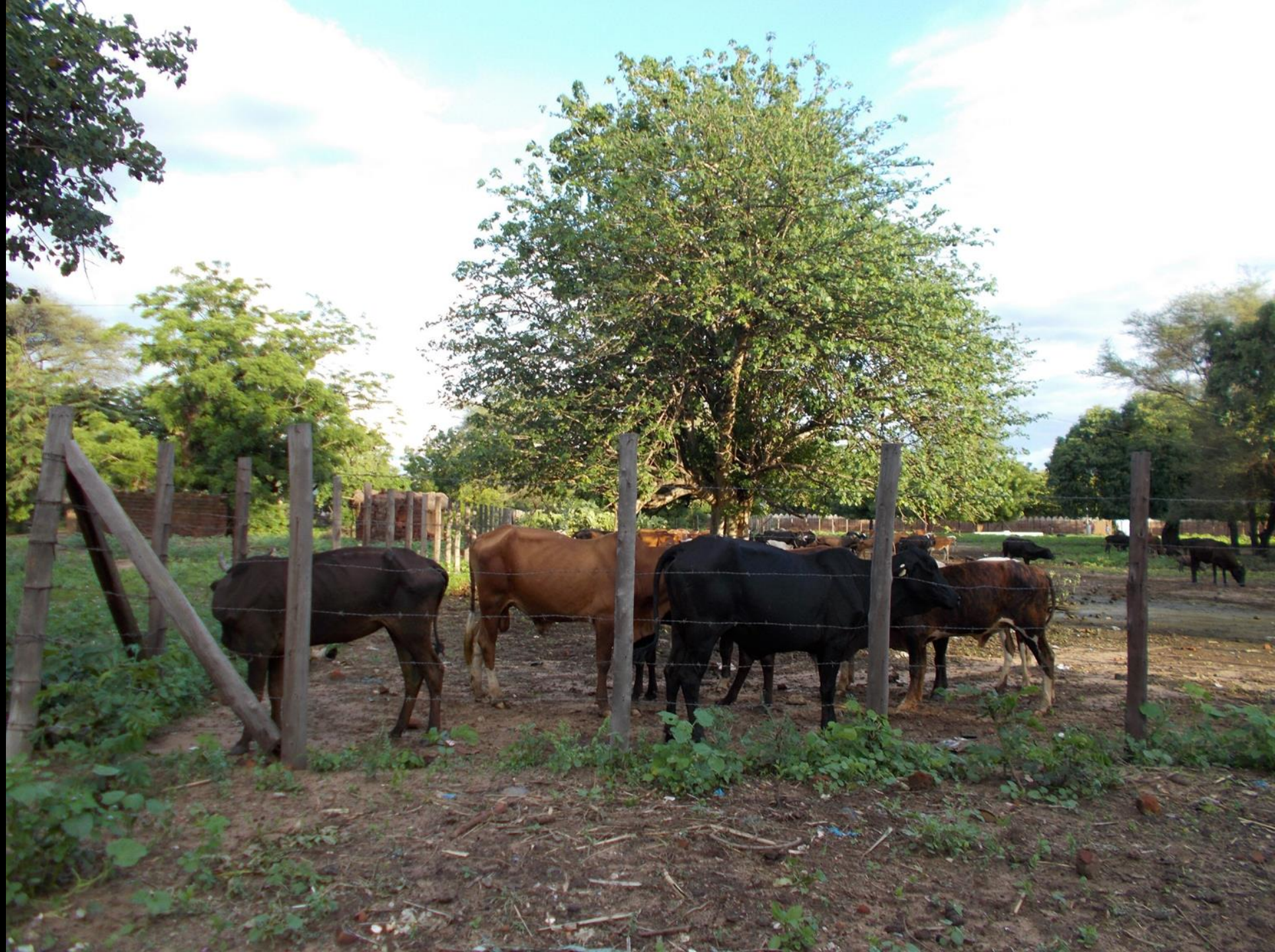

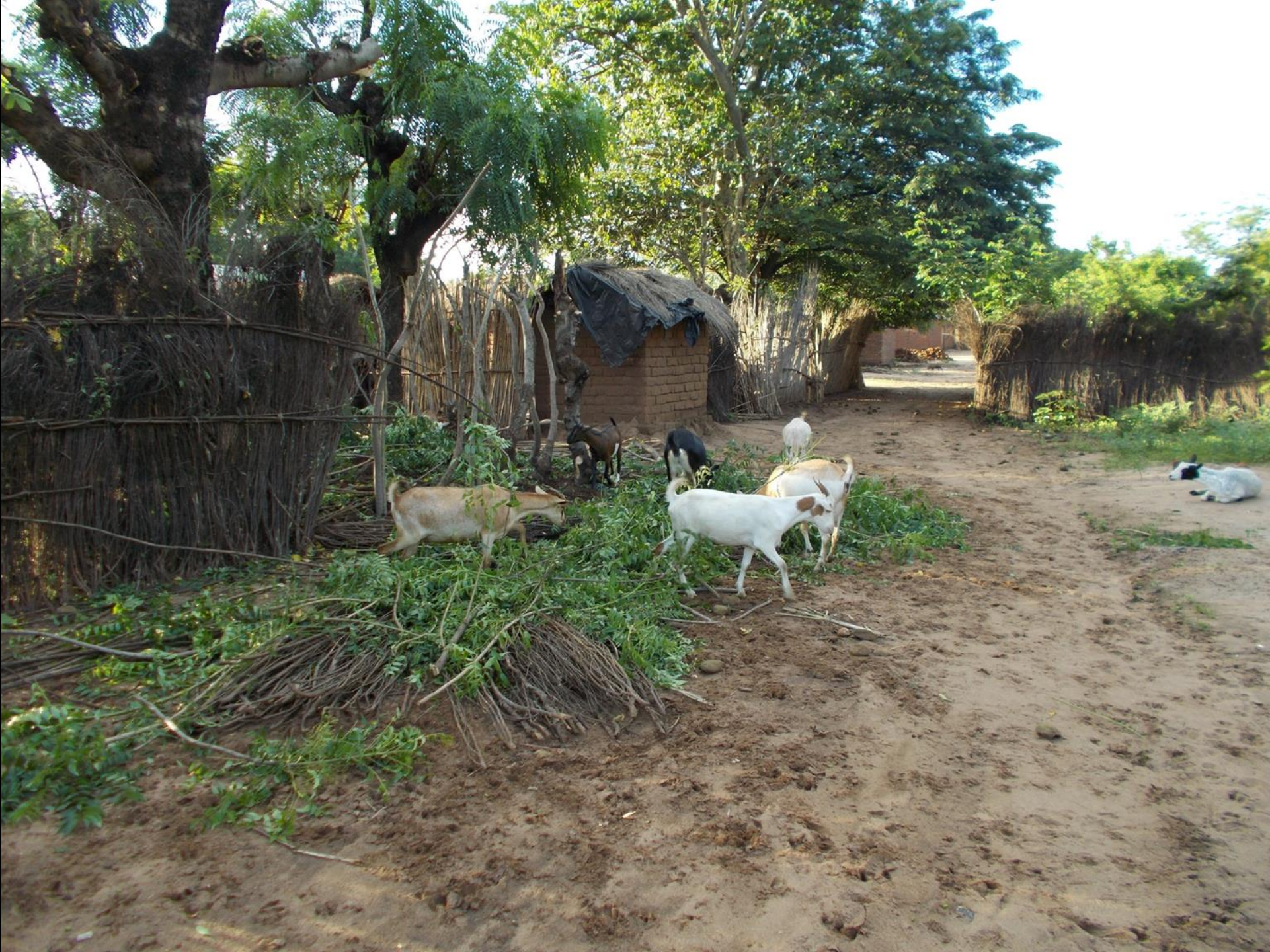

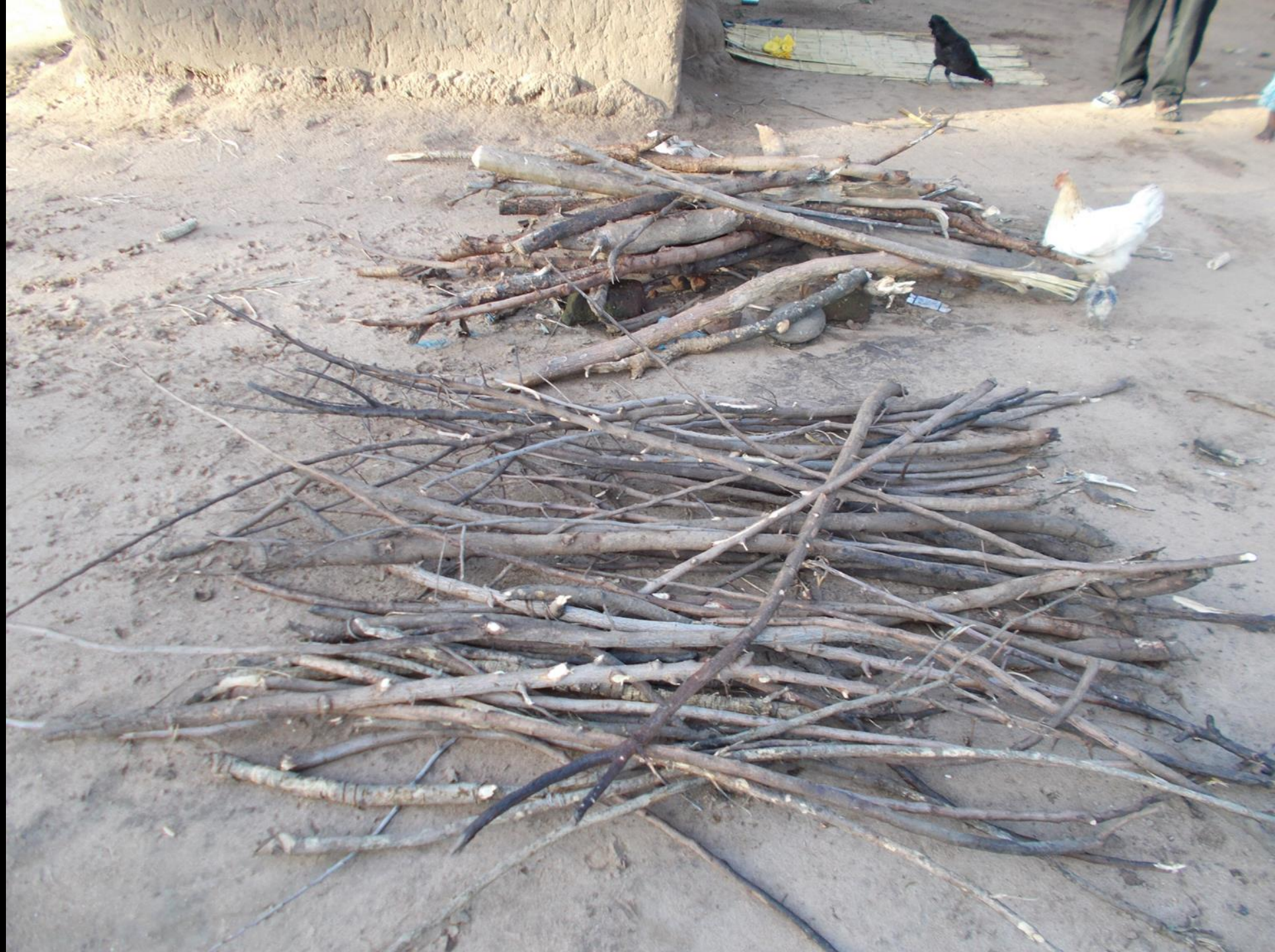

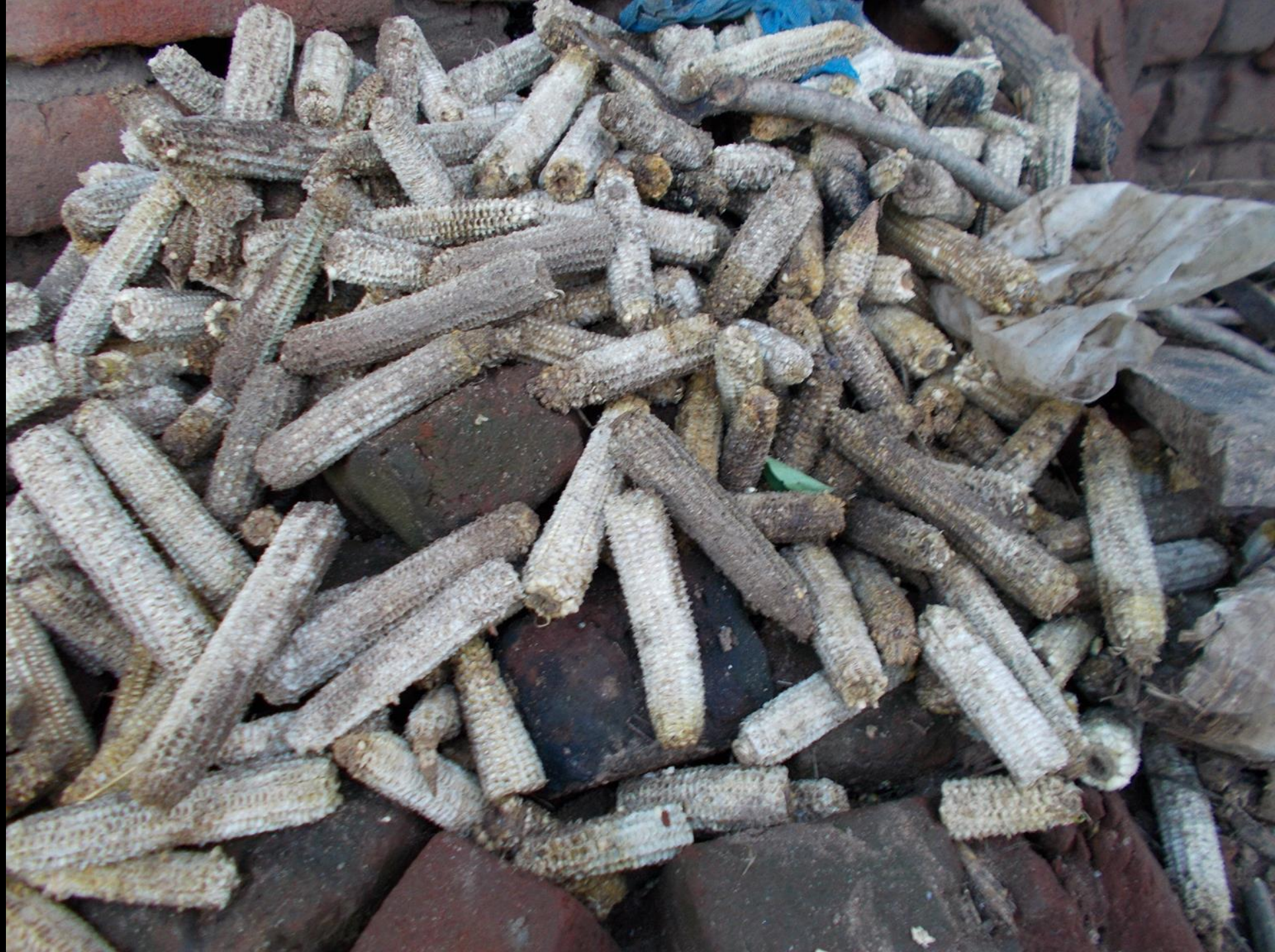

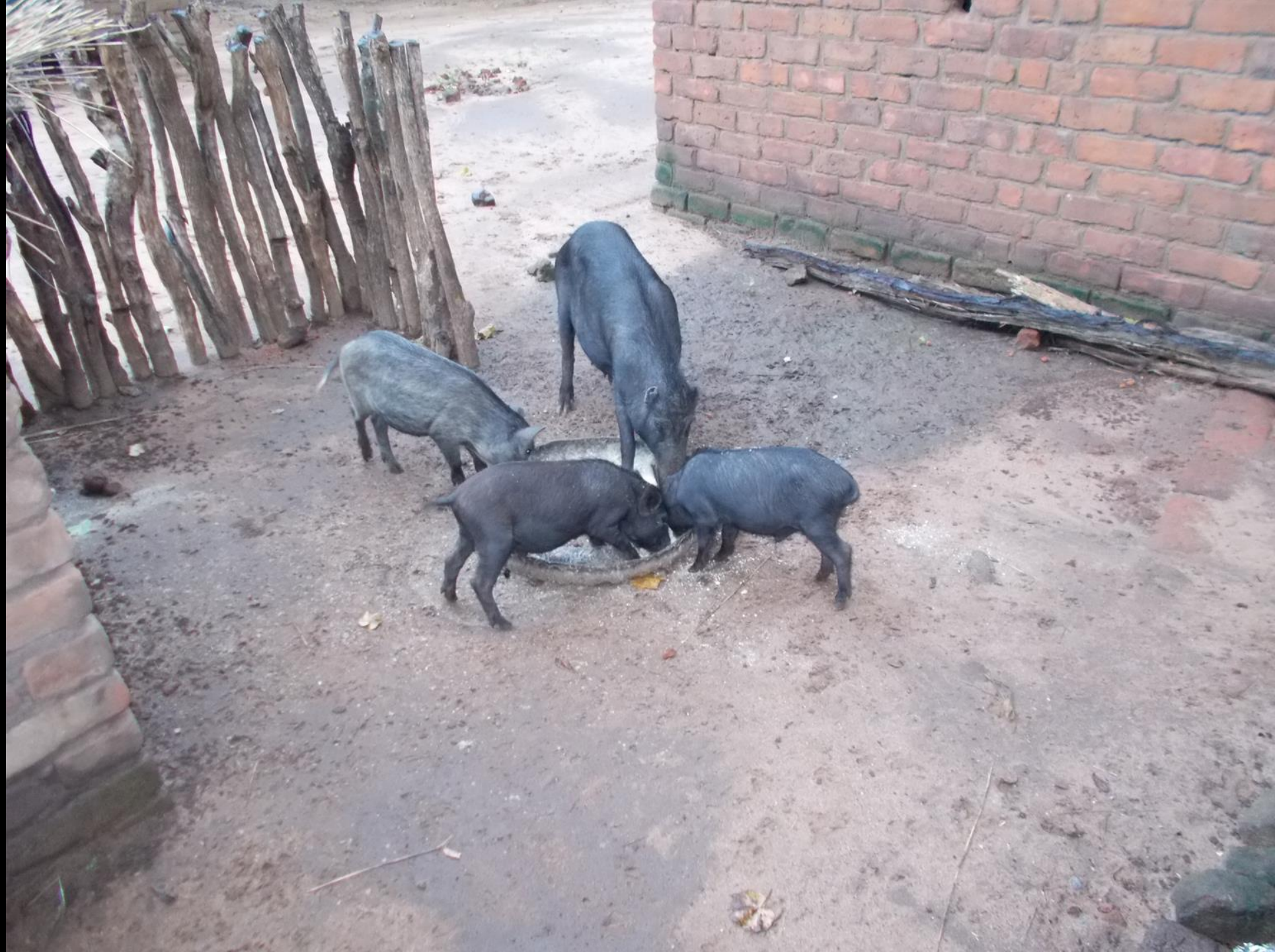

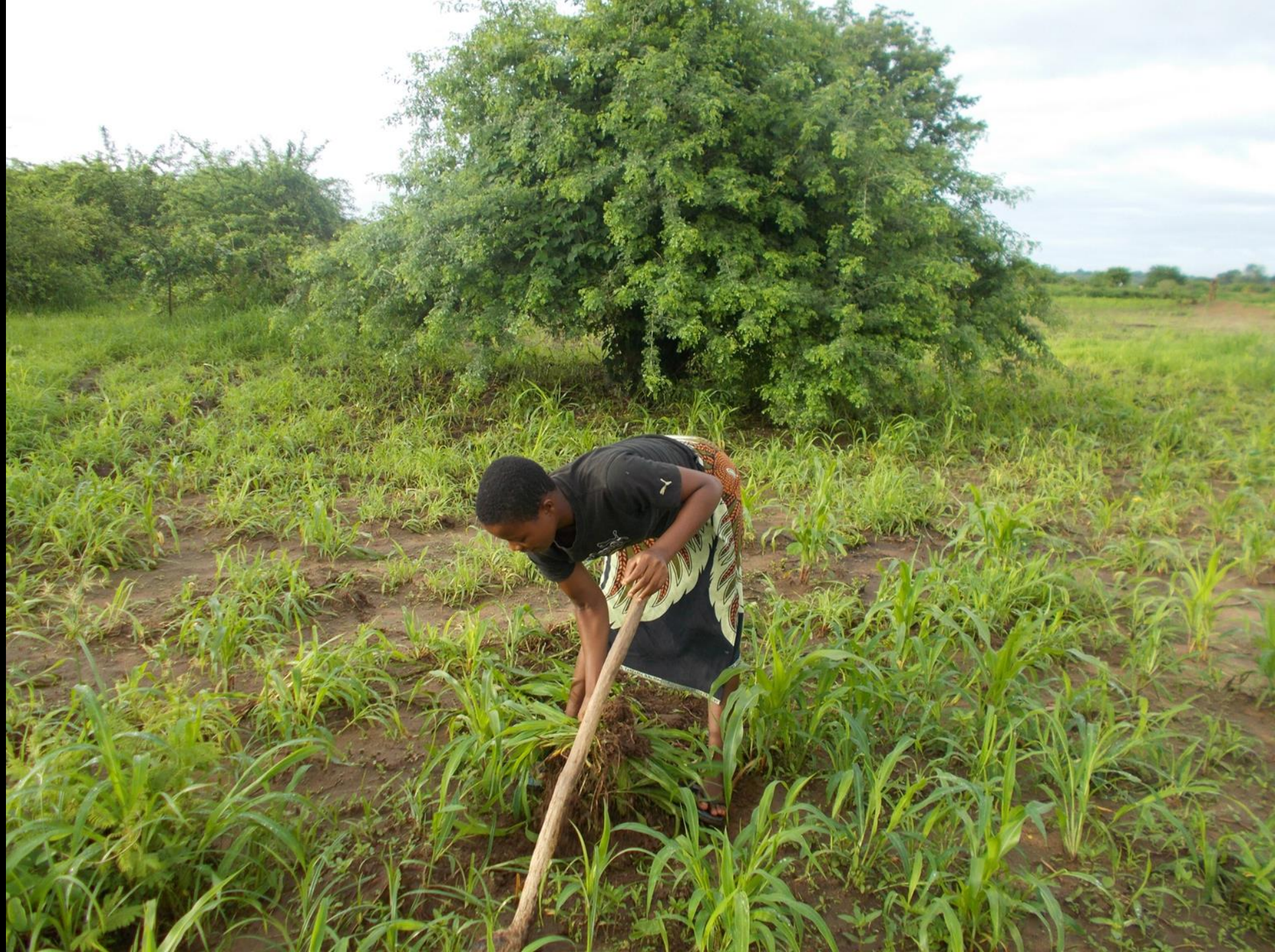

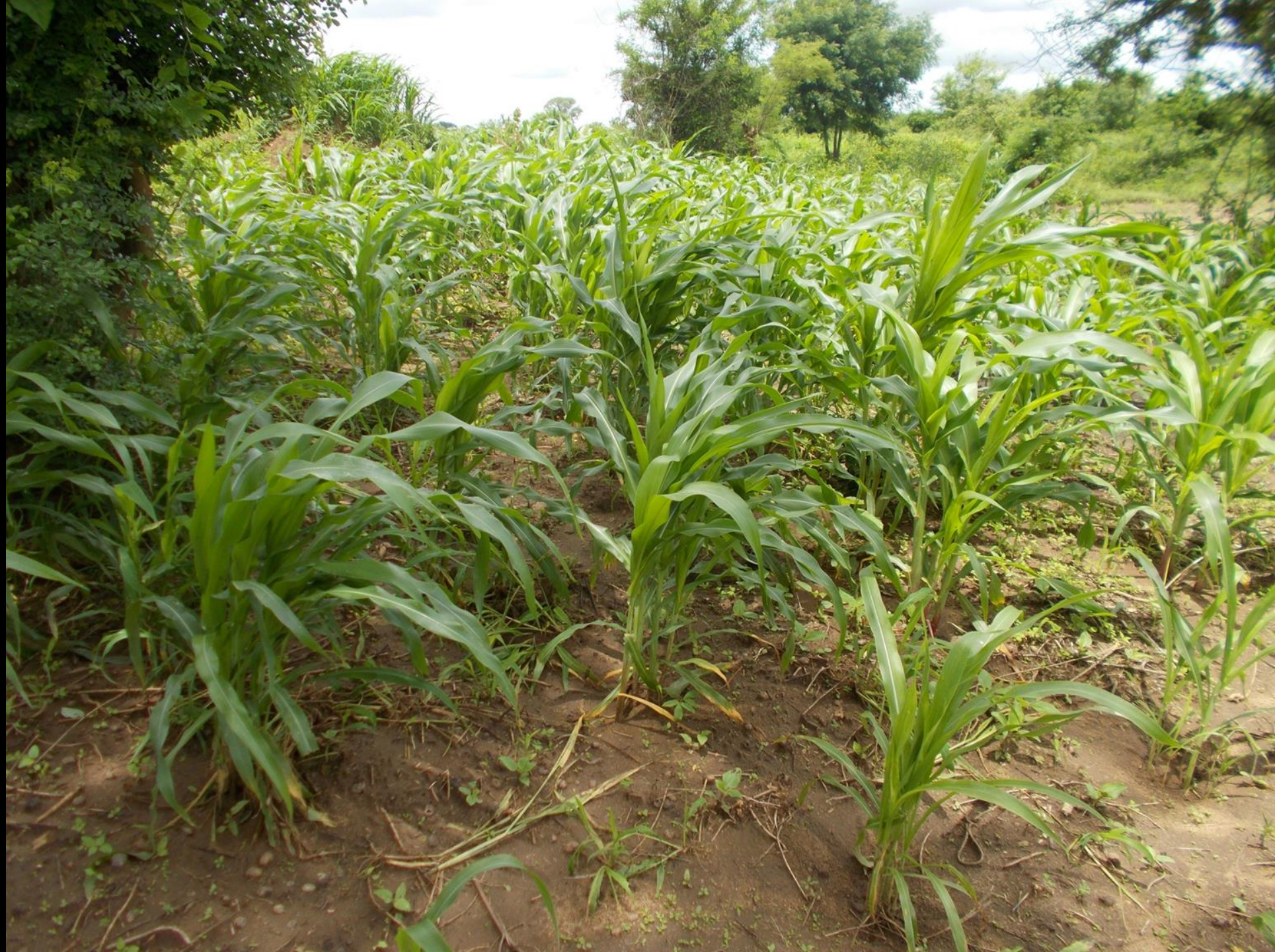

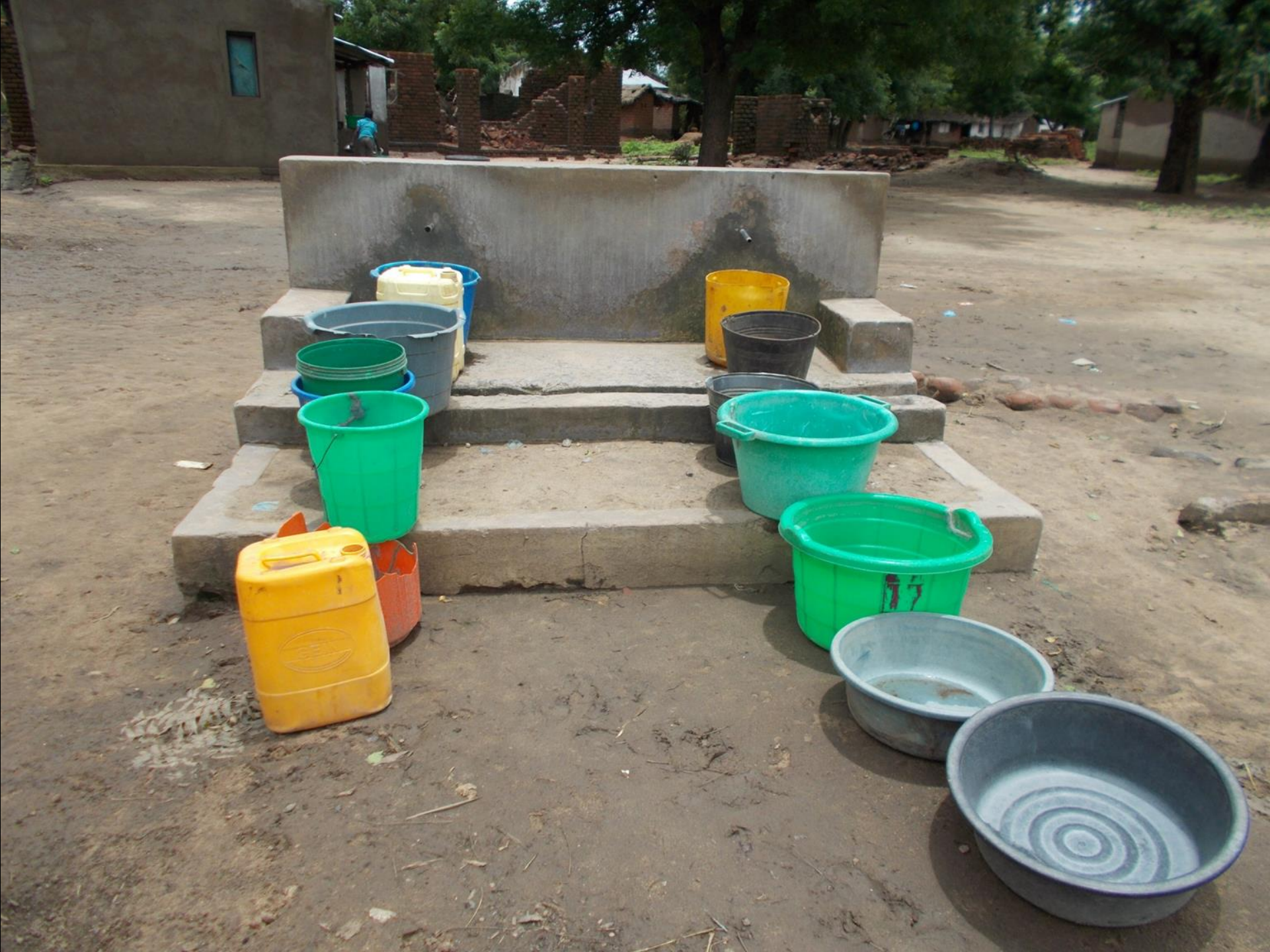

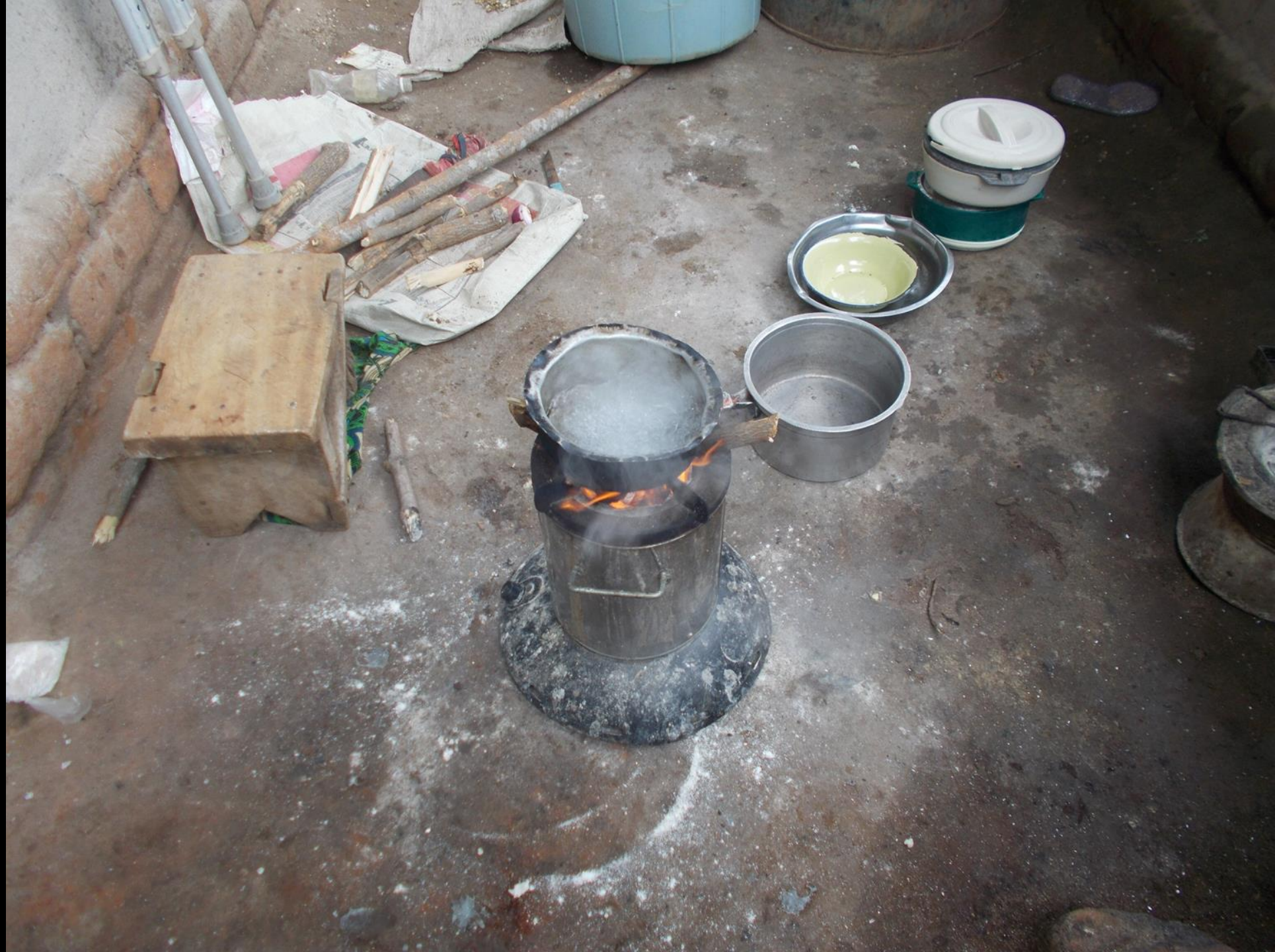

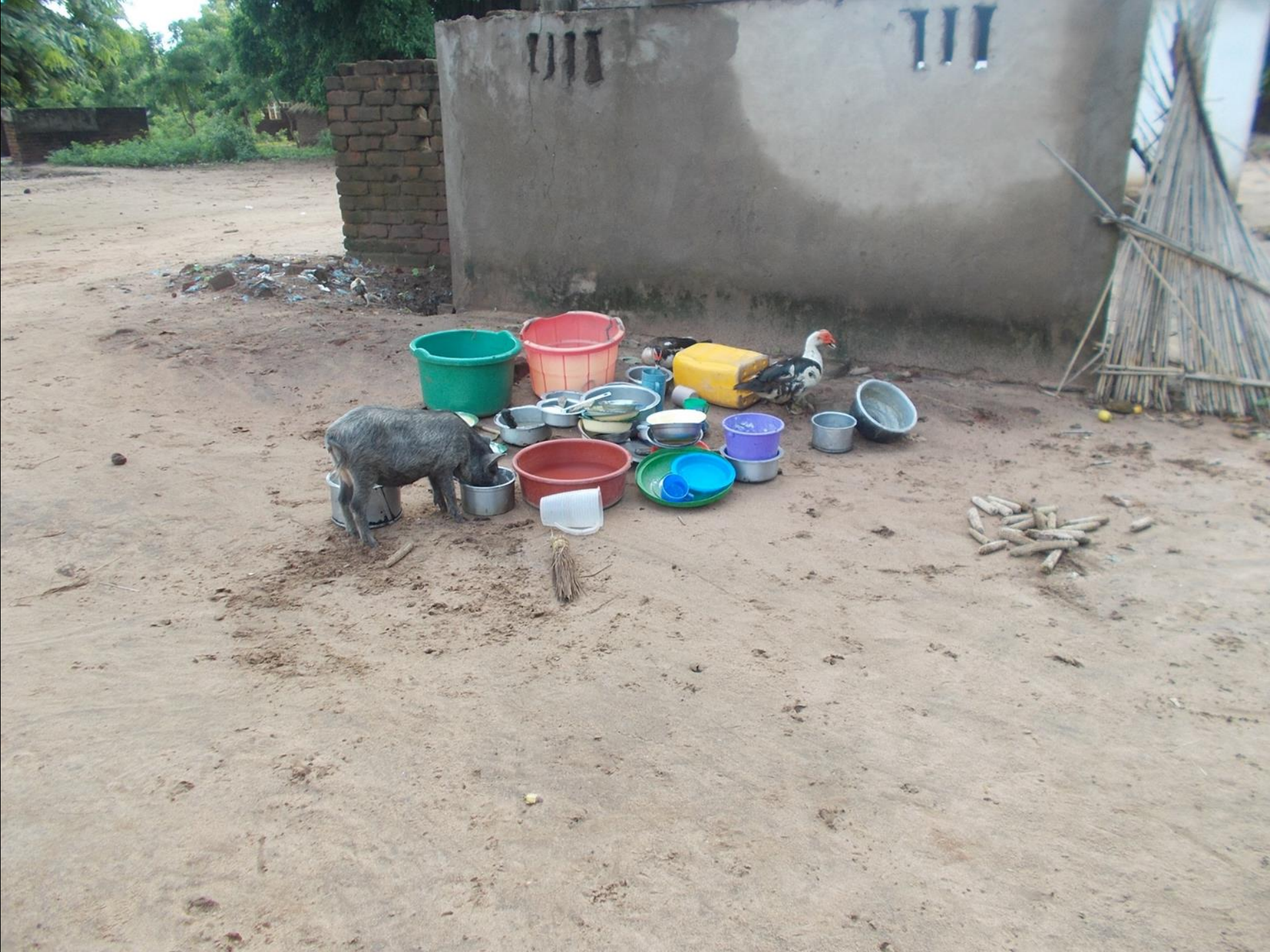

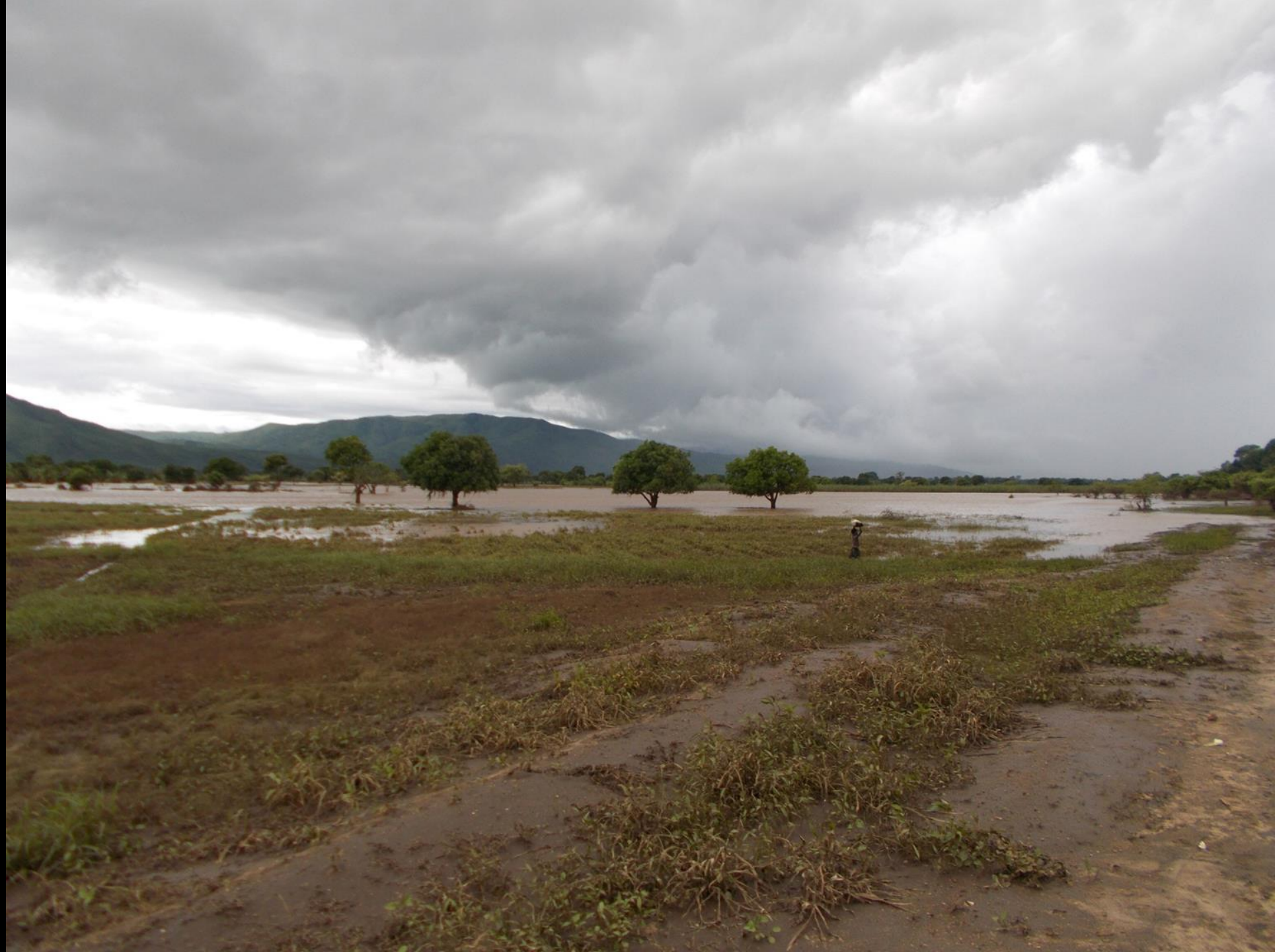

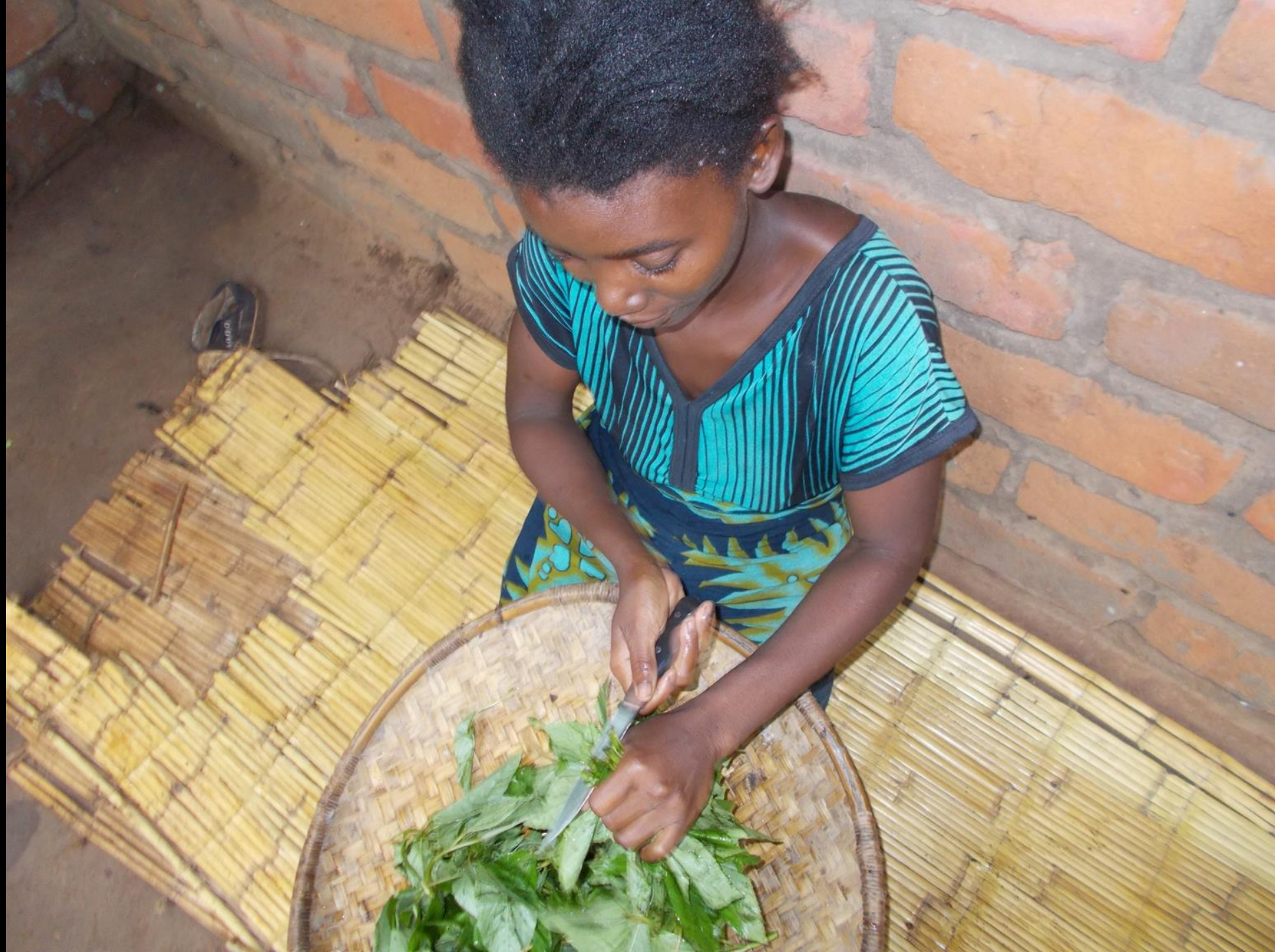

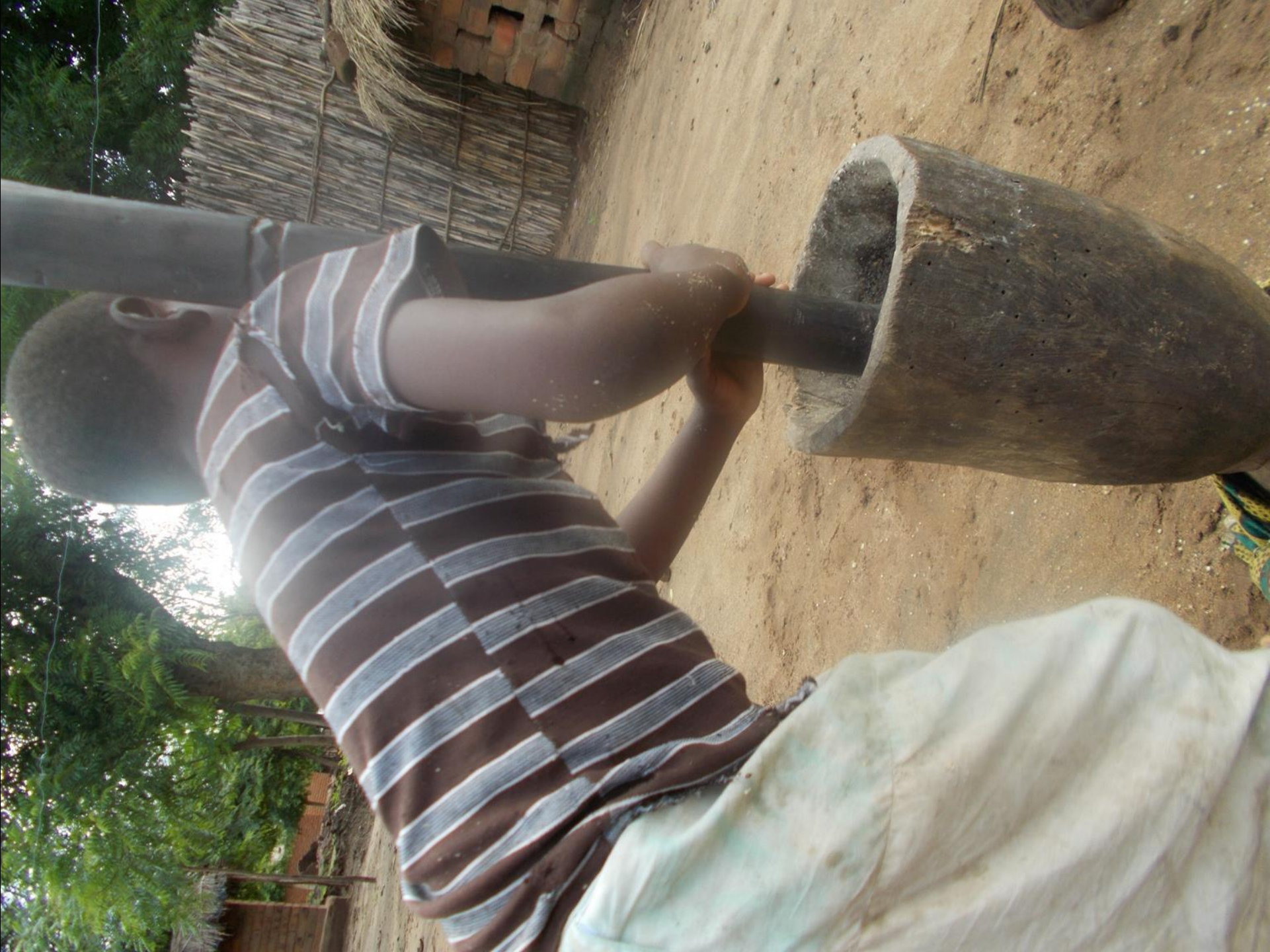

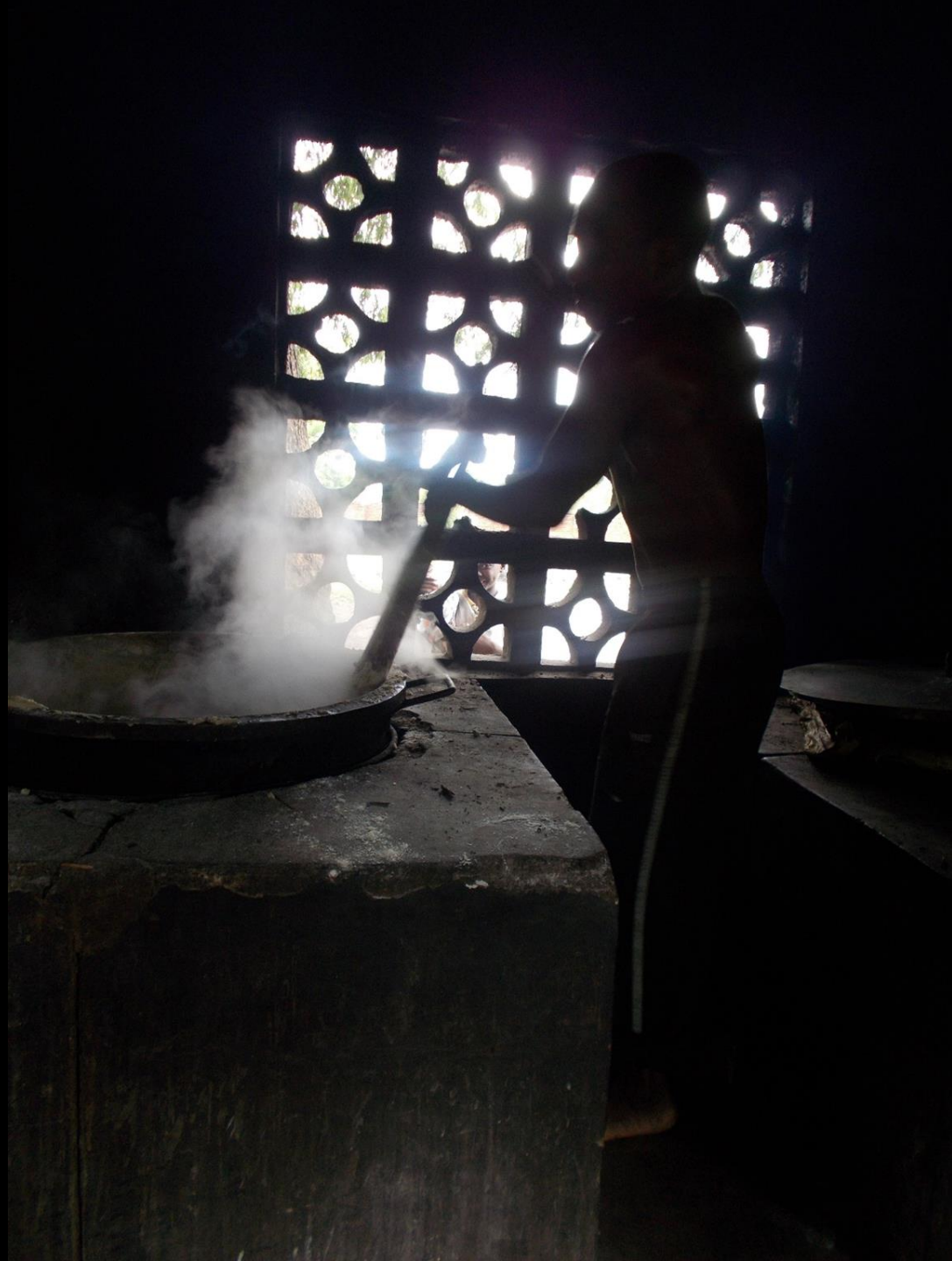

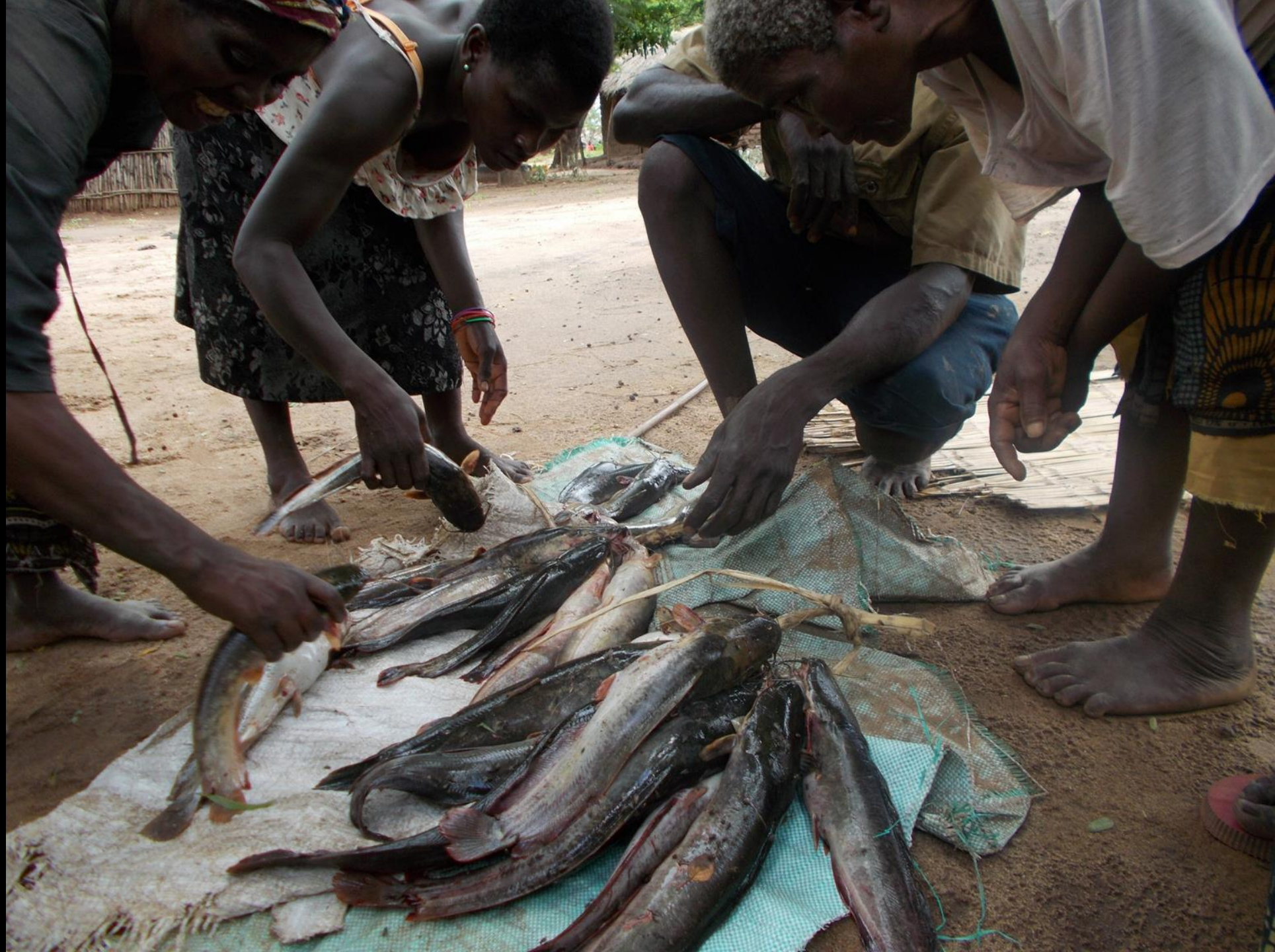

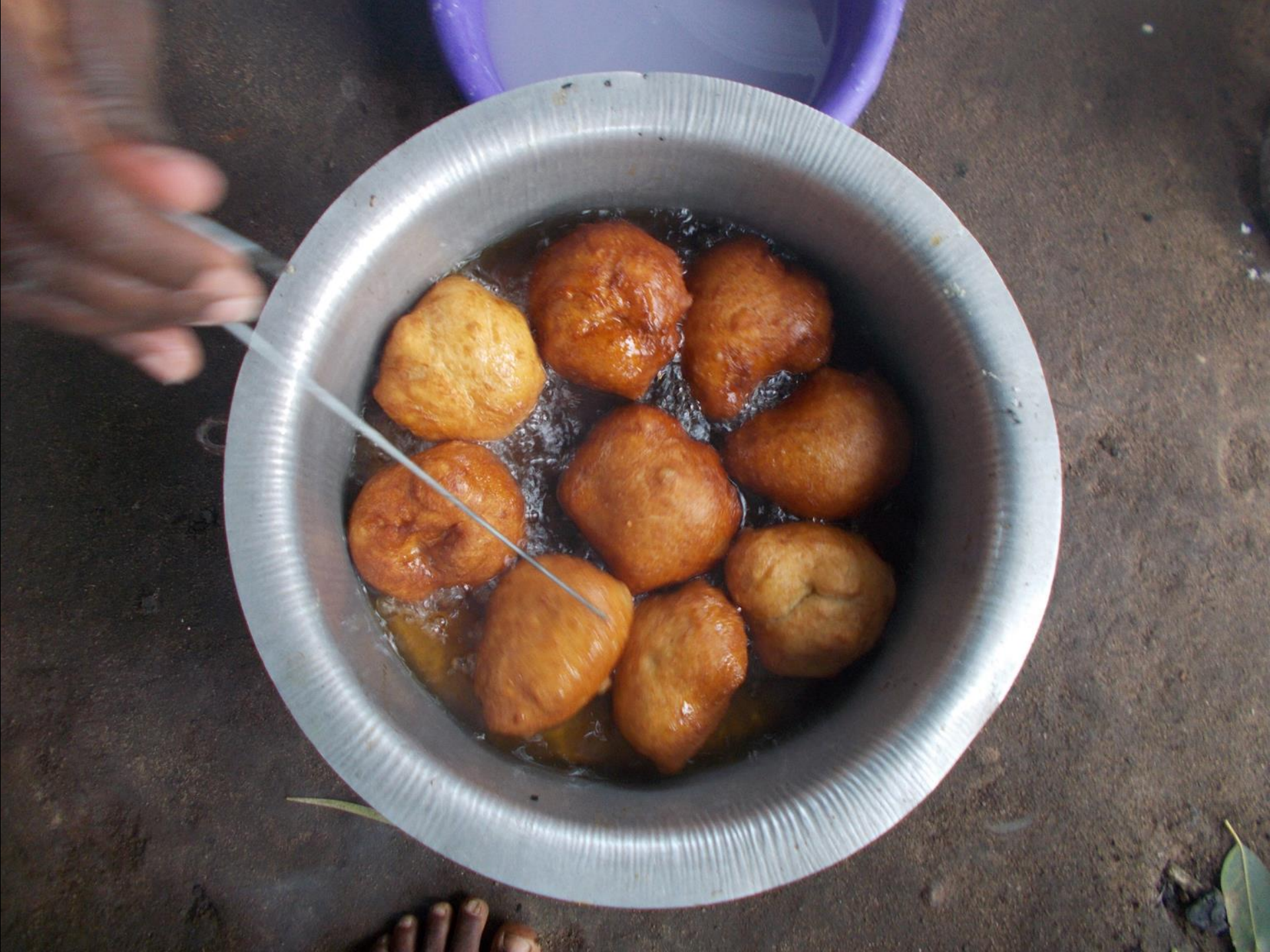

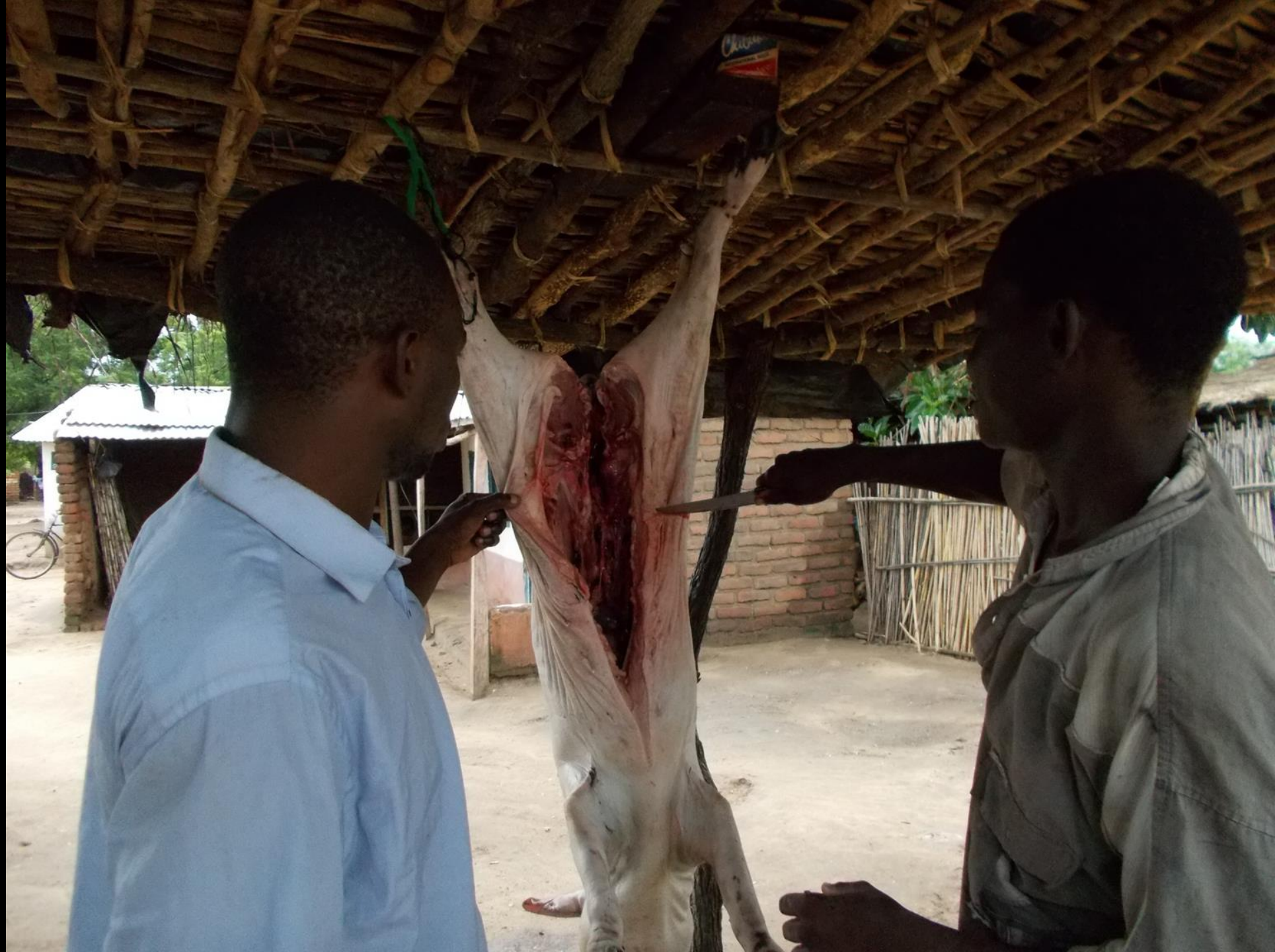

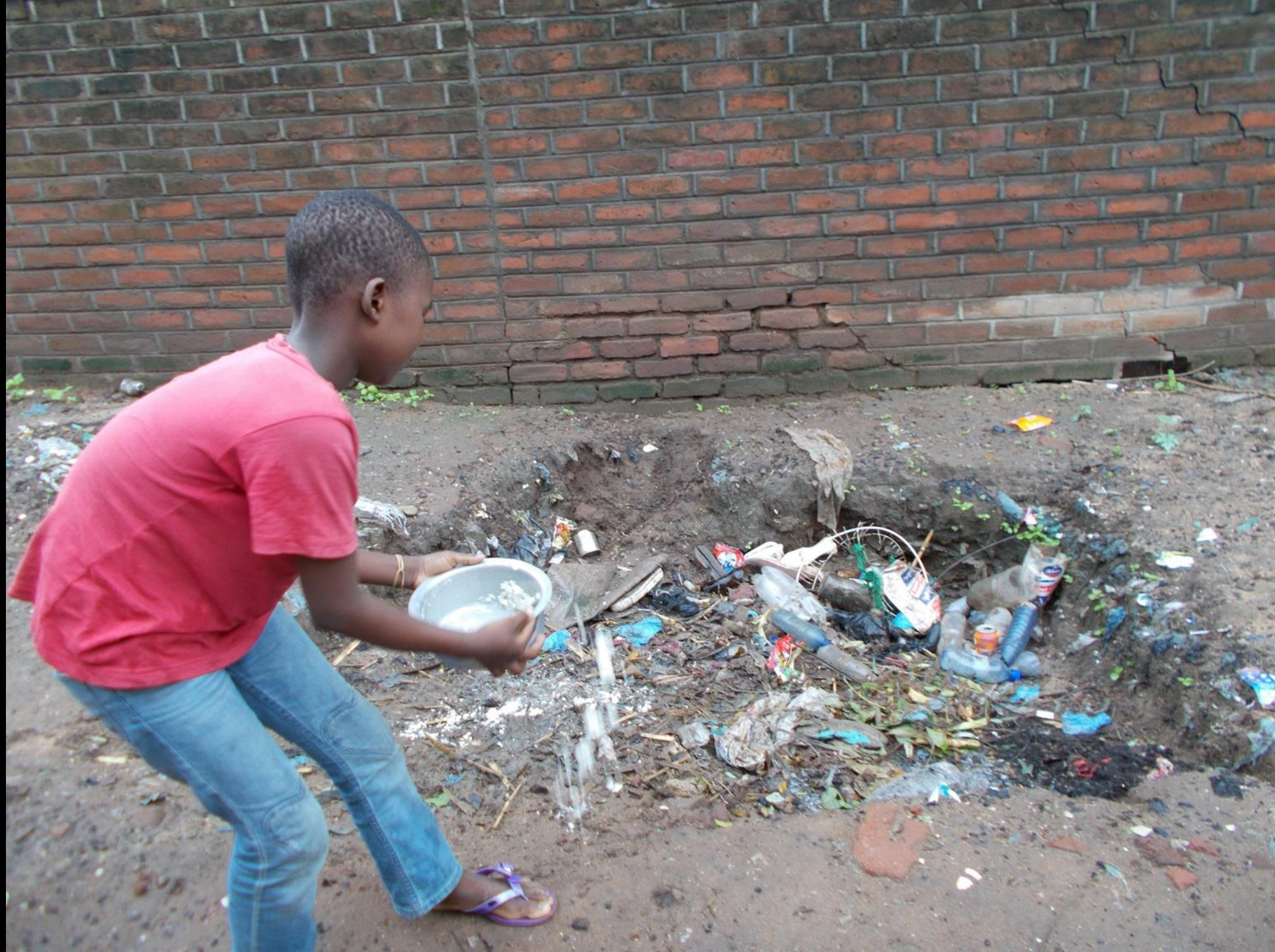

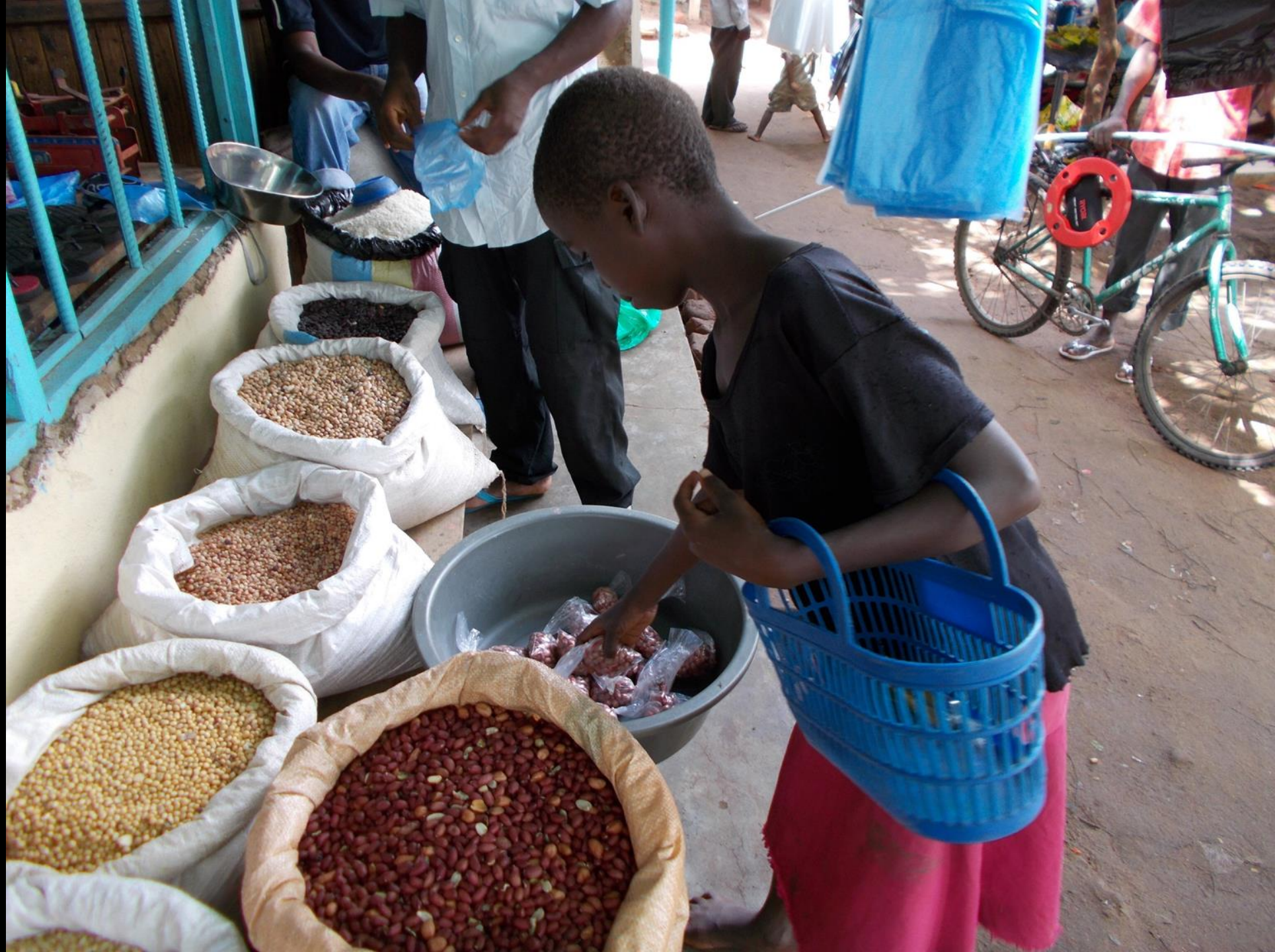

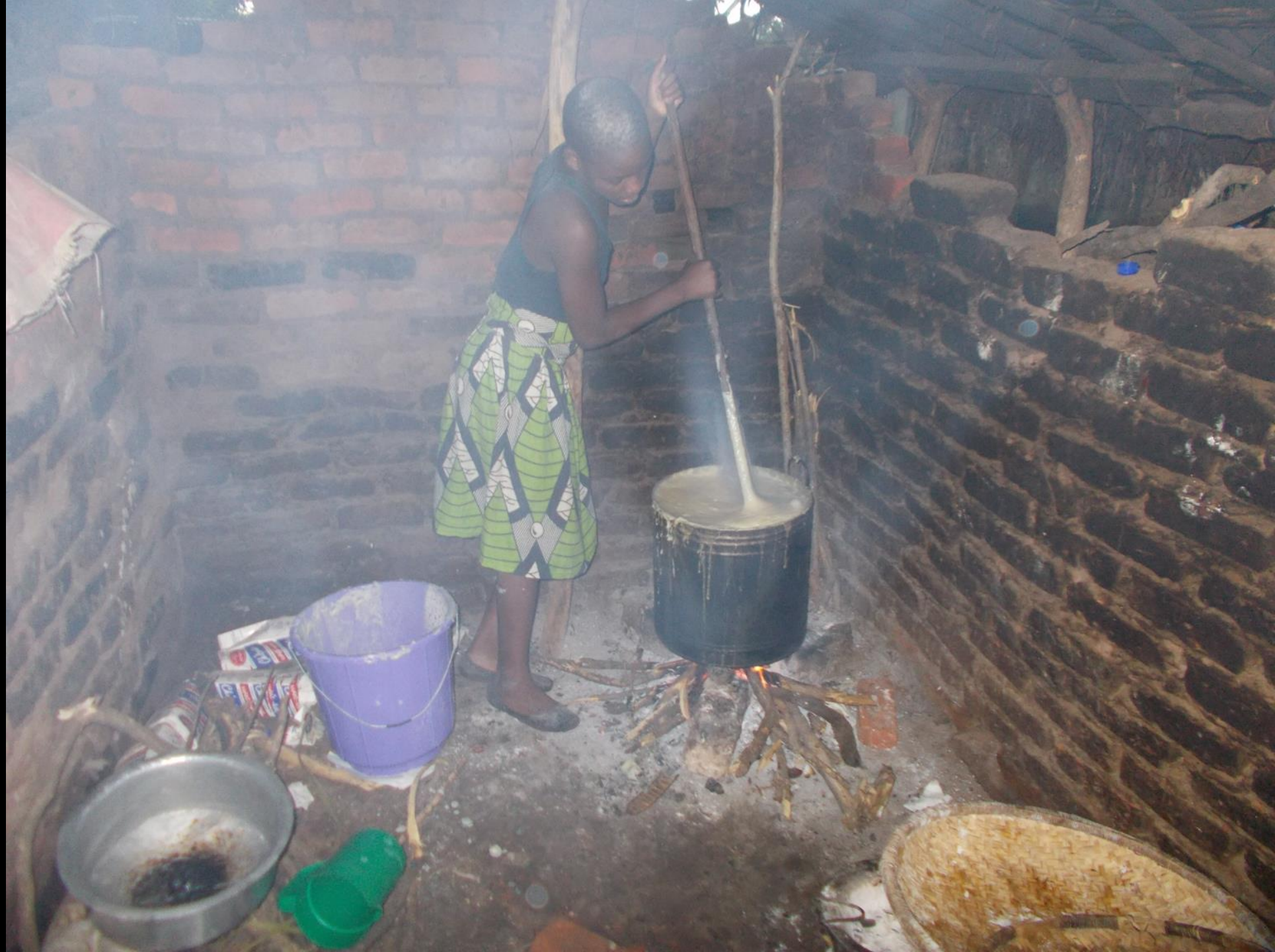

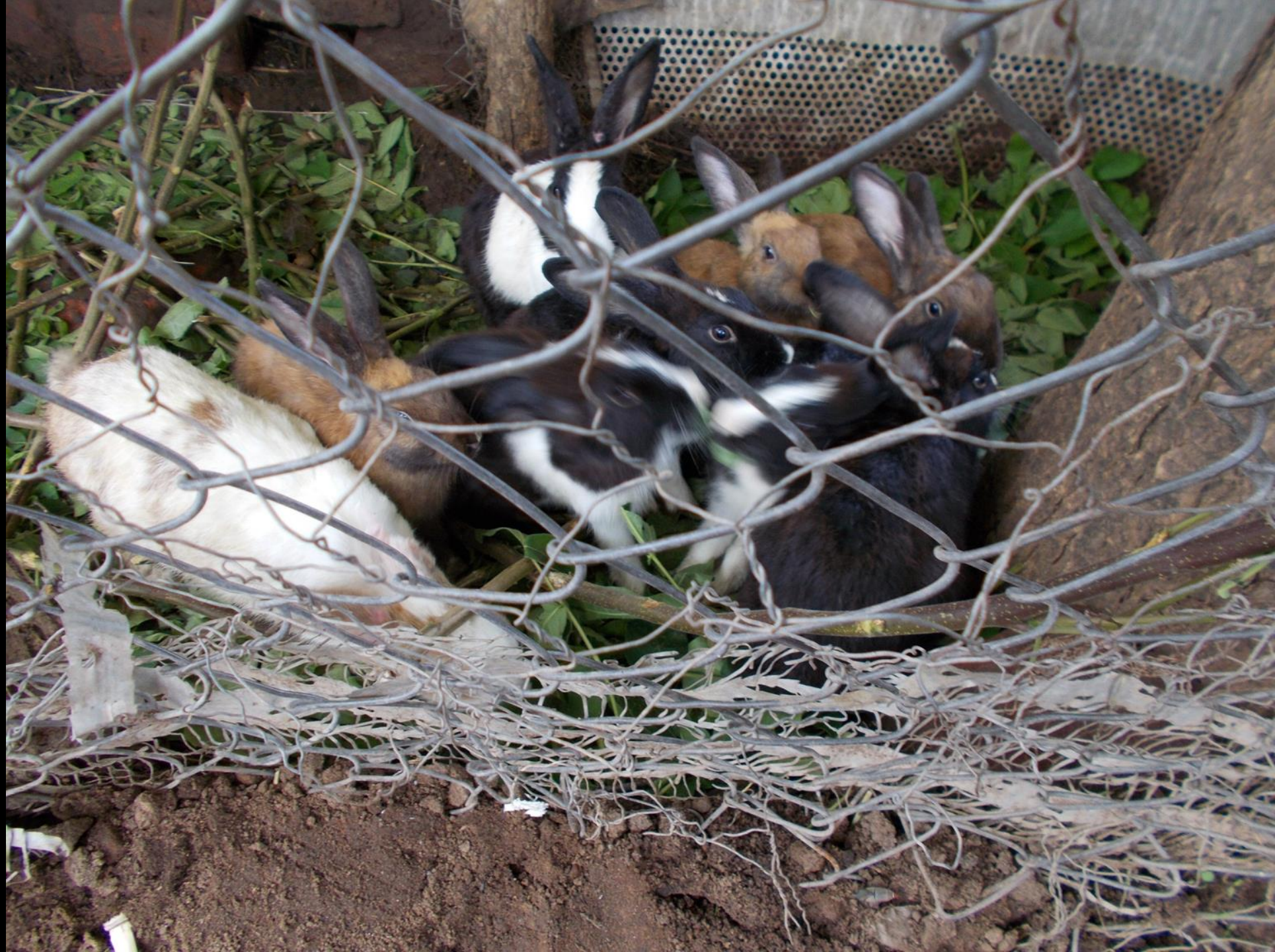

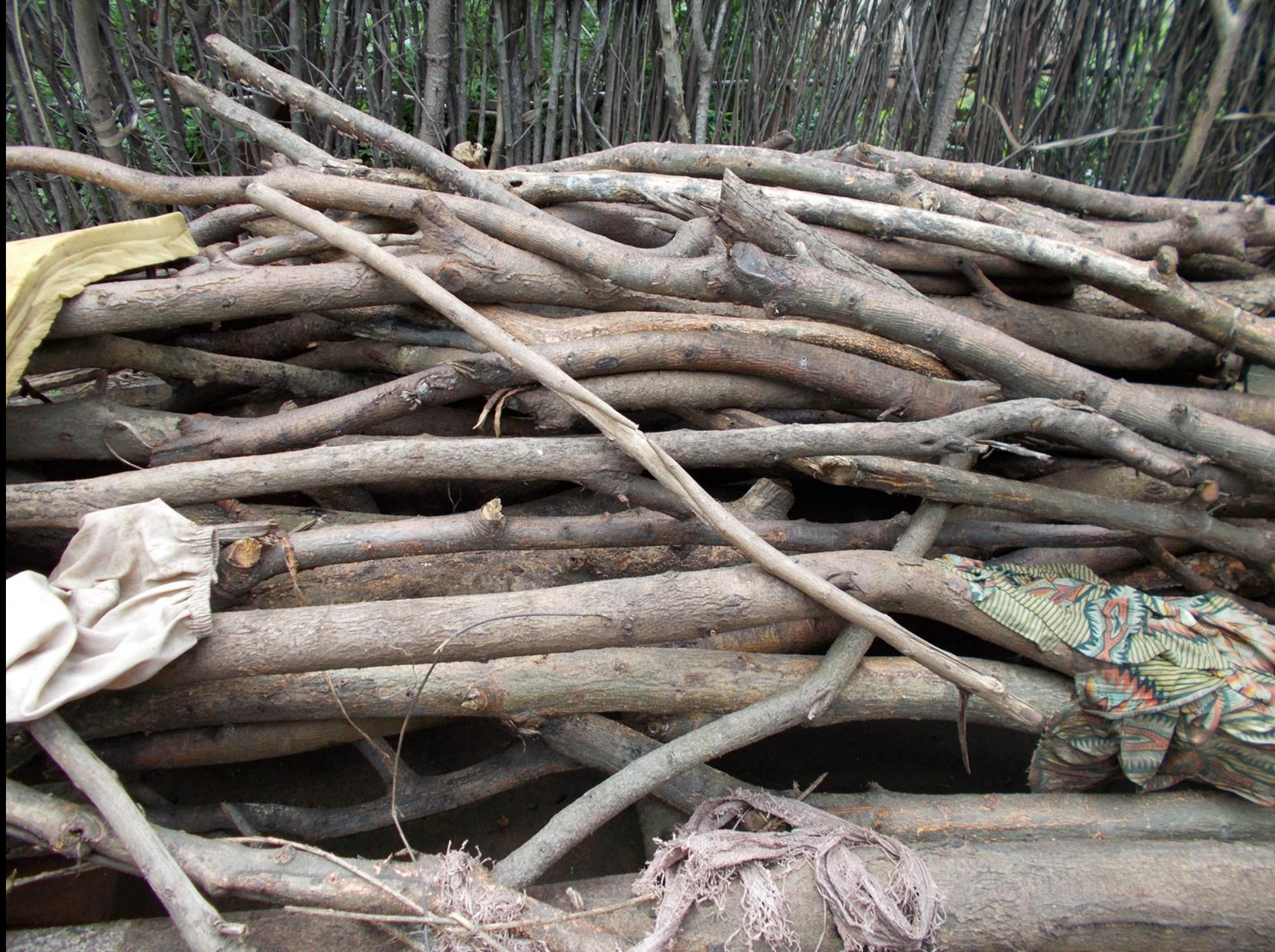

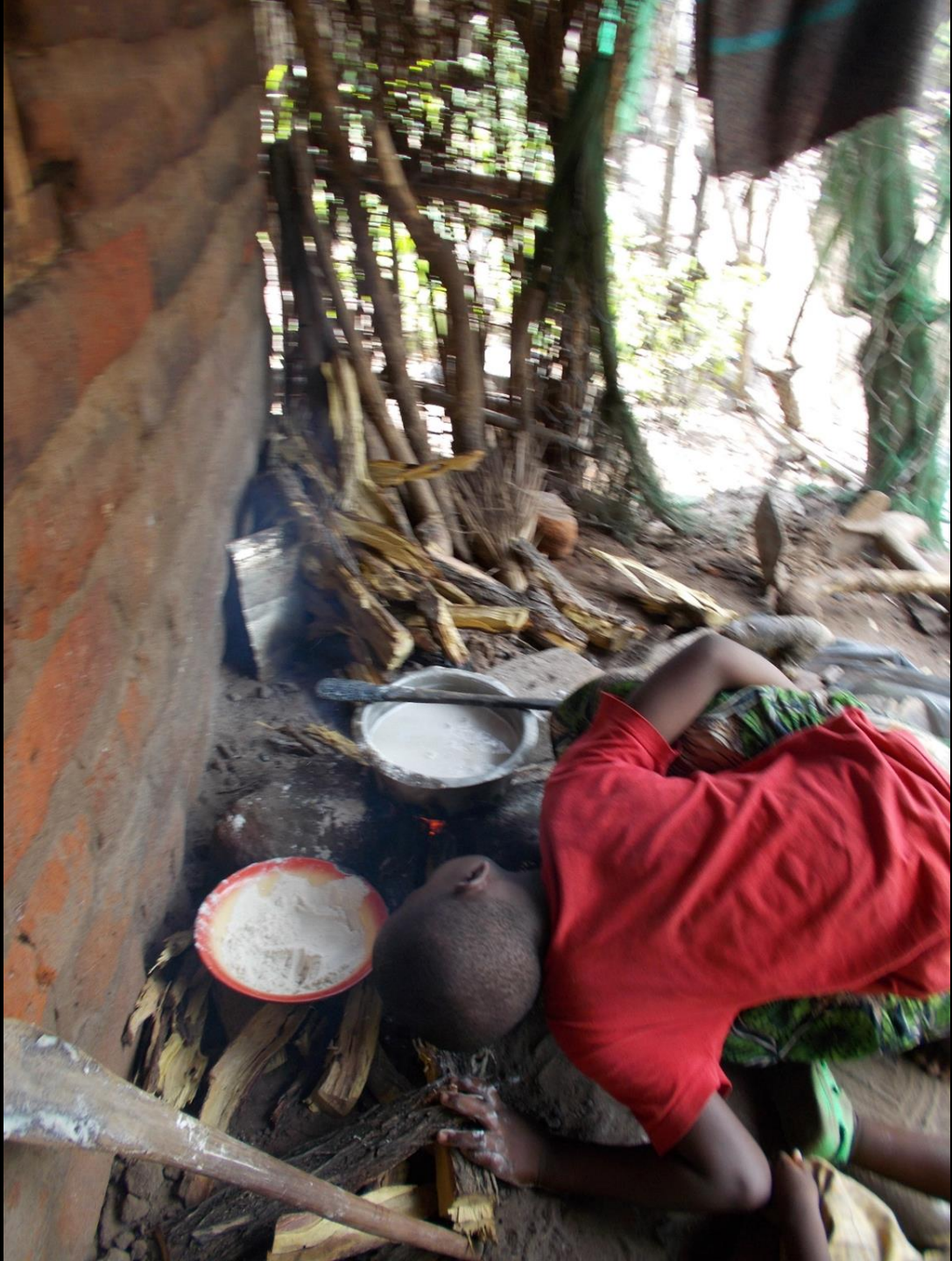

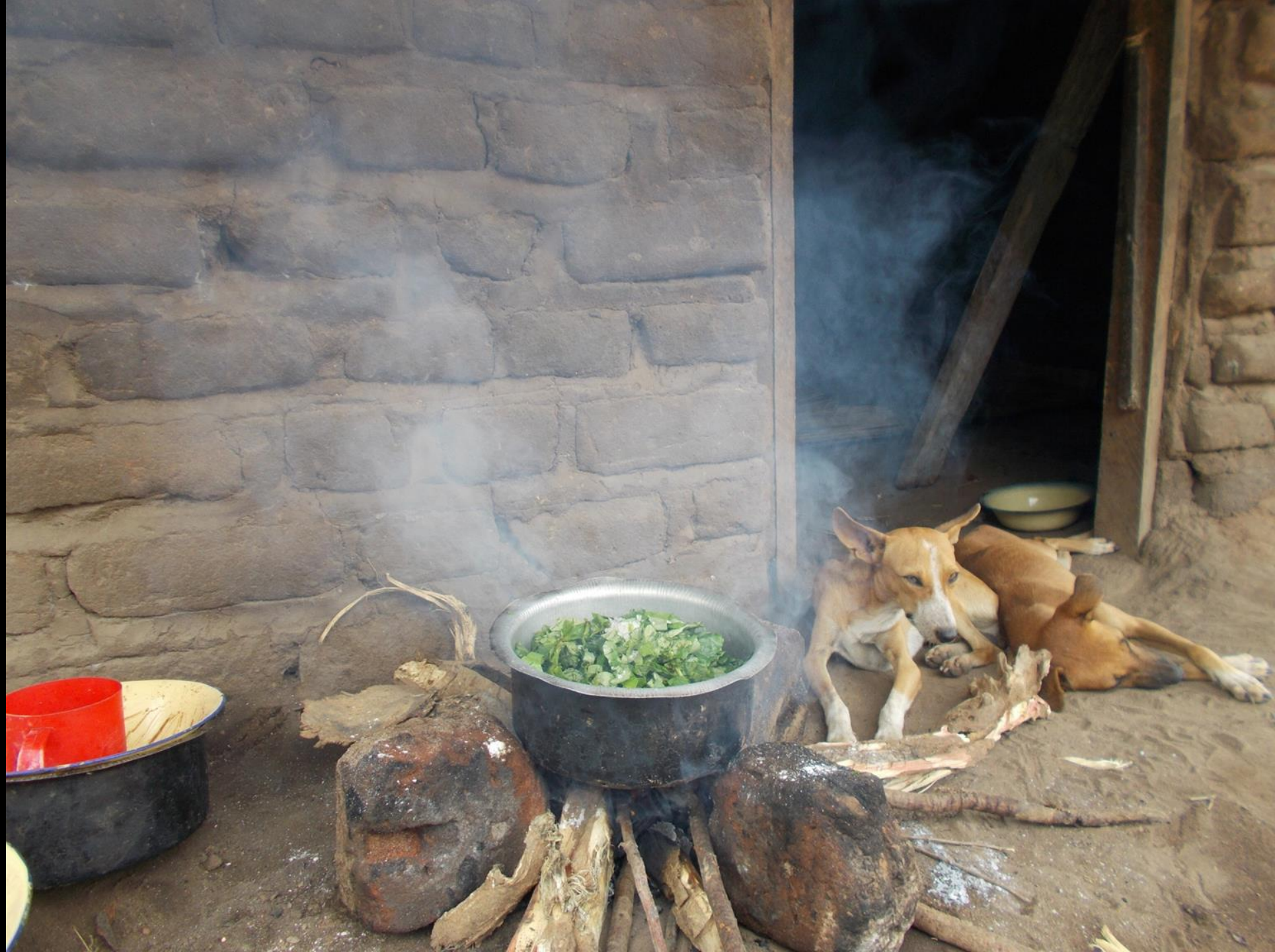

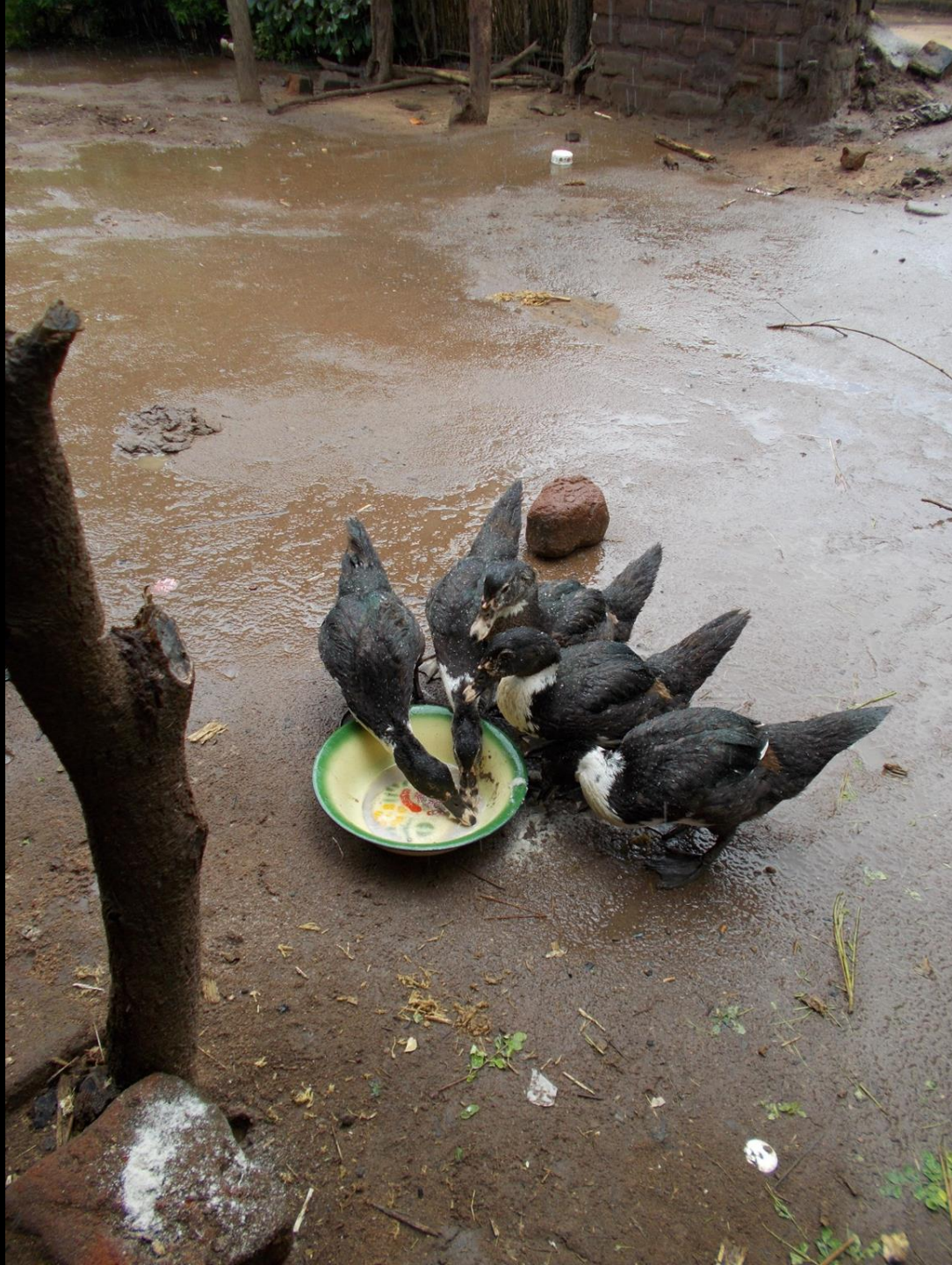

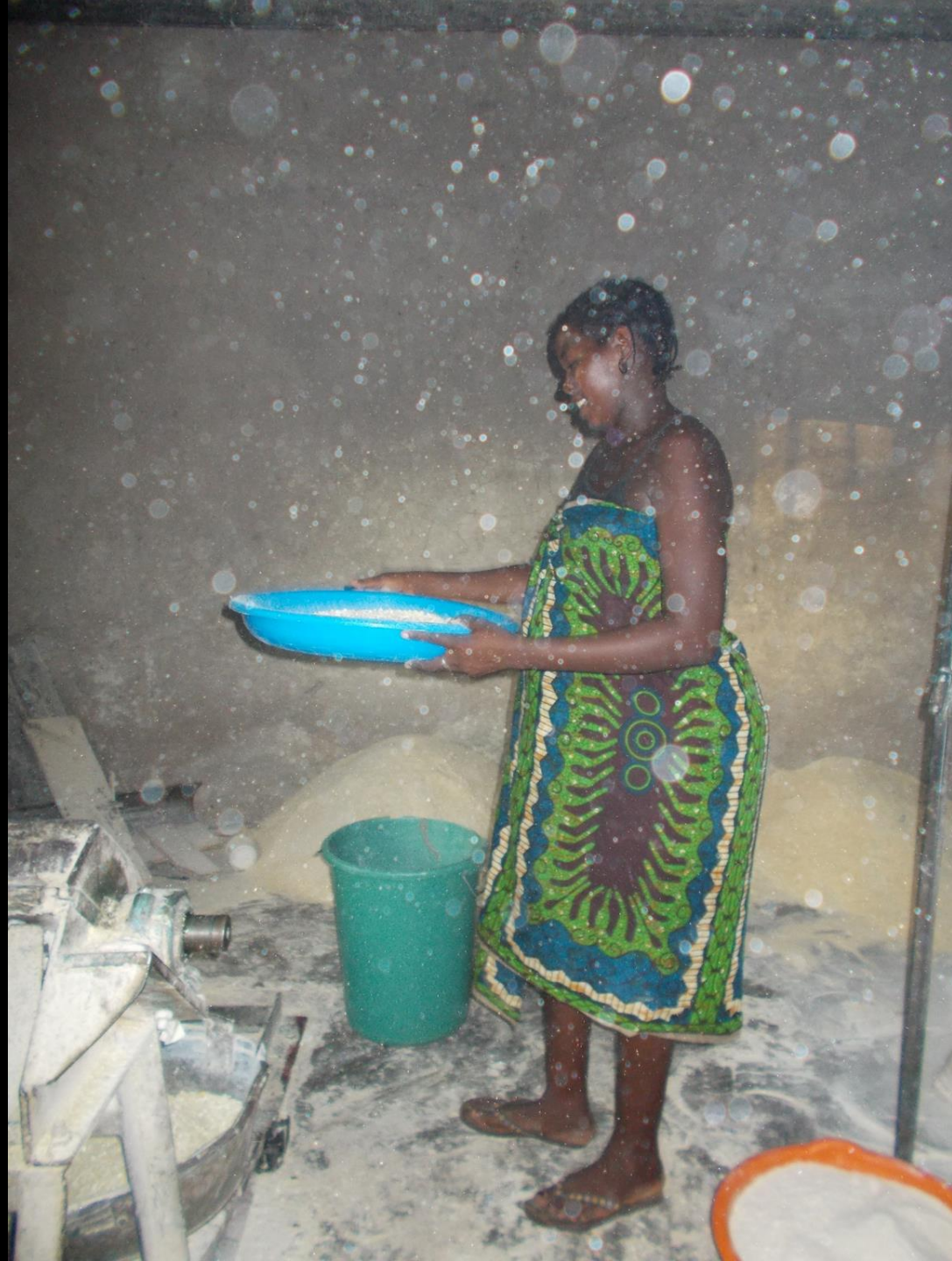

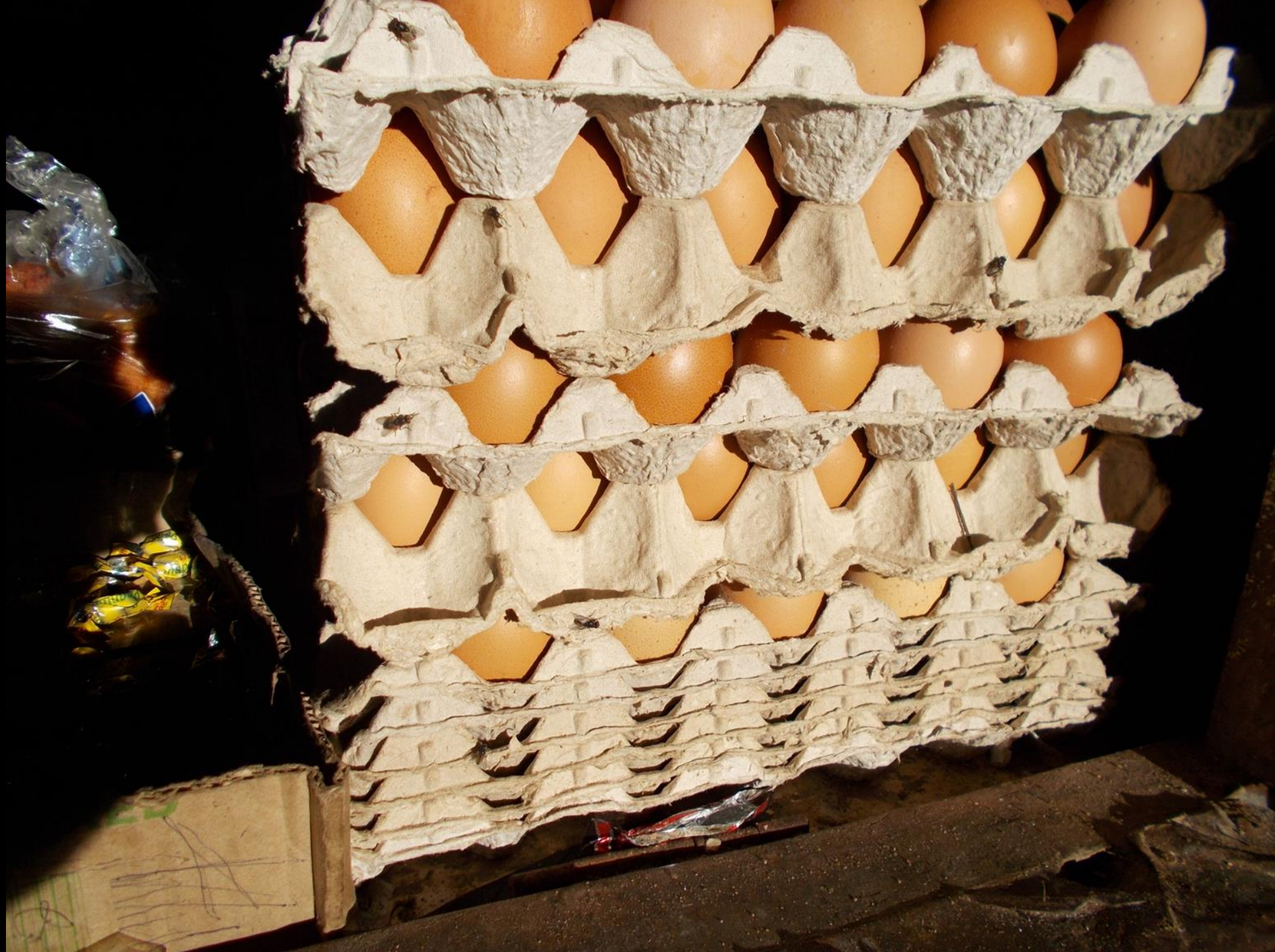

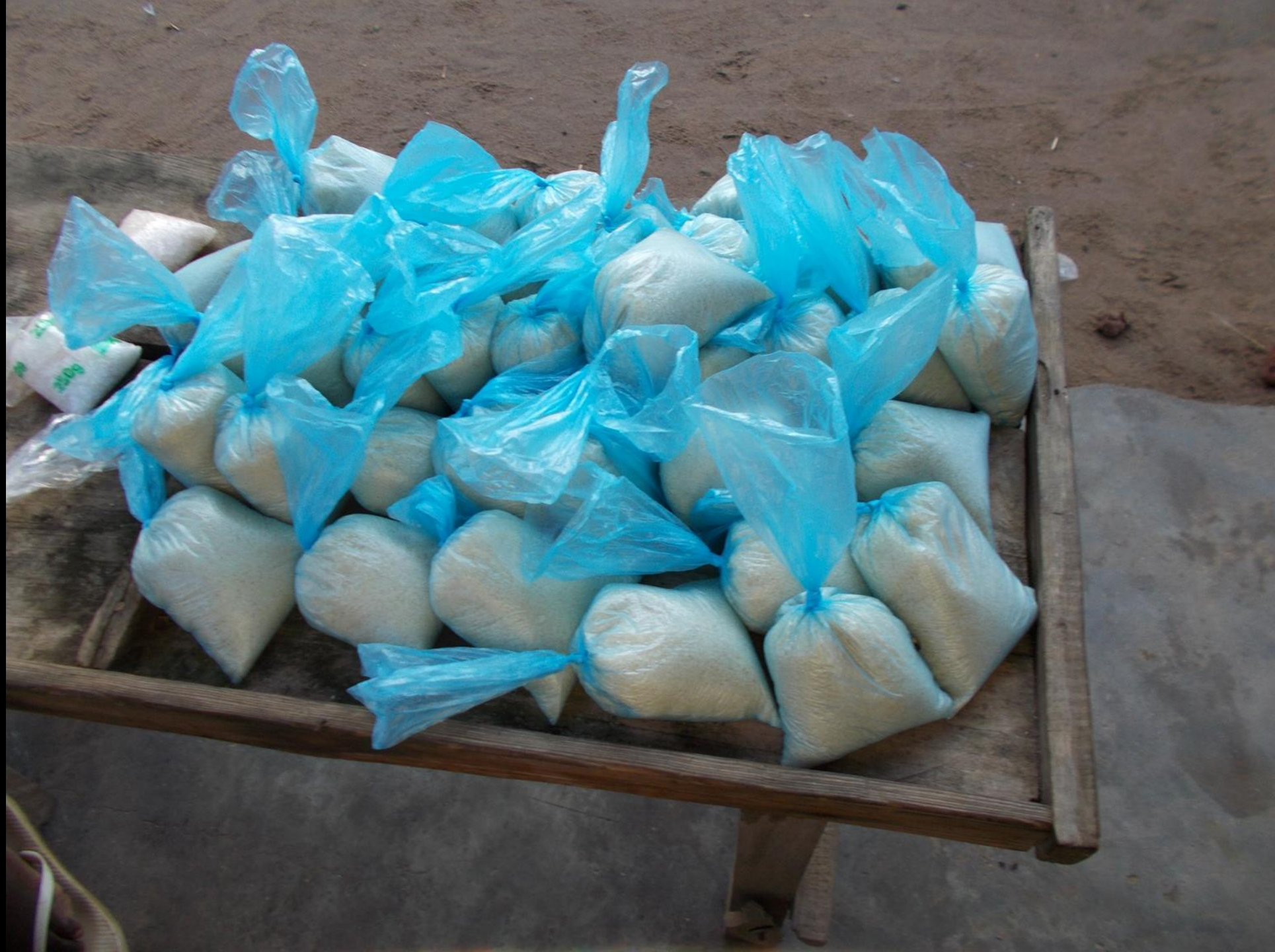

Supplement: S1 File — (PDF) [file pone.0156500.s001.pdf]
