# Supplementary material for: The Cooking and Pneumonia Study (CAPS) in Malawi: A Nested Pilot of Photovoice Participatory Research Methodology
Source: PLoS One. 2016 Jun 2;11(6):e0156500. doi: 10.1371/journal.pone.0156500 (PMC4890783; doi:10.1371/journal.pone.0156500)
Supplement: S2 File — (PDF) [file pone.0156500.s002.pdf]

**Interview 1**		
00:00-35	FW1	Aaaah, thank you very much! After asking you to take part in this study concerning food, you accepted and starting from a few days ago, we borrowed you the camera, you went and took the pictures that are here but what is needed per every protocol is your consent that is accepting and signing that you are going to take part in this study.
00:36-44	FW1	So,here we have already written down your name, telephone number, address, do you have a box number so that we should also include? The one you use for receiving letters.
00:44-44	R1	Box number?
00:45-46	FW1	Yes
00:47-48	R1	I have forgotten, what is the box number for Codecom?
00:48-49	R1	its 162 right?
00:49-49	CHILD	162!
00:49-50	LADY	Yes
00:50-54	FW1	Box care of 162,52?
00:54-57	R1	162
00:57-58	FW1	That's ok.
00:59-31	FW1	On this page I will ask you to write on your own declaring that you are allowing MLW to use photos, videos or words that have been recorded….2015, right!
01:32-00	FW1	Who did you choose to be your witness? So that he/she is required to sign. You should sign here and write today's date, its 17.
02:00-17		Background noise…
03:18-36	R1	My name is (R1), I am marrried with two children but we also stay with my elder sisters daughter. My husband works in Mozambique as a driver.
03:37-46	FW1	The children in your house including the two of you are of what ages?
03:47-56	R1	The other one is fourteen, the other is five and the last is two years and months.
03:56-04	FW1	Are these the only people you cook food for in your house or there are also others?
04:04-09	R1	These are the only ones but there are also other people who come from other houses, sometimes we eat with them.
04:09-11	FW1	How many are they?
04:11-13	R1	About three.
04:13-36	FW1	From these pictures try to explain those ones you can on what you were doing at that moment. You can arrange them properly and let us know what message is conveyed in each picture.
04:43-58	R1	This one shows us how we grow crops. The crops are well taken care of until the time of harvest and then we get them home.
04:59-17	R1	This picture is also showing us how crops are taken care of at the farm. The trees are cut so that light reaches fully on the crops. On the other hand, the picture is also showing us that the trees cut when clearing the fields can also be used as firewood at home for cooking food.

05:43-03	R1	There, that picture shows a charcoal stove that is used when we have no firewood for cooking, we set fire using charcoal in the stove as an alternative.
06:03-34	R1	This boy is sweeping outside their house. This picture shows a group where this boy realizes that it is necessary to clean the premises so that when his mother is cooking, the premises should be clean to ensure that the food is not contaminated with various diseases.
06:34-57	R1	These two kids are defeacating on a latrine pit close to the place where they dwell and their parents use the same place when cooking food as well. The faeces produced by the two kids are bad and those two animals next to them are eagerly waiting to make food out of it when the boys are done, an act that is health hazardous.
06:59-16	R1	This picture shows a Kraal and a house. The kraal is used to store food and crops when we don't have sacker bags after harvesting.
07:16-48	R1	This is a church, a place where we go to thank God for giving us food. For us to get food it's the power of God through rain and also for us to work up healthy ready for work and find strength to cook.
07:49-59	FW1	Can you be a little bit clarify on what is your staple food?
08:00-02	R1	Nsima and relish.
08:02-06	FW1	Which crop do you process this nsima from?
08:06-08	R1	From the maize we grow.
08:08-10	FW1	Only maize?
08:10-12	R1	Yes and millet
08:12-13	FW1	Only?
08:15-30	R1	Yes, sometimes we use sorghum that is relatively smaller as compared to millets, we use them to make nsima when millets and maize are scarce.
09:30-33	FW1	Ok, please hold on a bit.
09:30-33	FW2	Give instructions to respondent 2 (not very clear what was said)
00:25-00:29	FW1	Tell us more about food, drinks and how you prepare it
00:43-01:51	FW1 &	Should we stand or kneel? Yes, we need to kneel down. So, lets not mix them. Those from the garden should be in their own group and so on.
02:05-02:22	FW1	Explain in detail what we are seeing in all these group starting with group one. What message is this photograph telling us in terms of food?
02:23-03:18	R1	The first photograph is showing us a location where we collect firewood for cooking our food. Sure. The second photograph shows the garden where we grown crops and the effects of the floods that was and destroyed our crop. The third one shows the cattle that we rare. These cattle do provide us with milk, meat when we eat and also cash when we sell during the time when we need have no maize for food we do sell these cows for cash and we in turn buy food.
02:19-03:42	R1	This sixth photograph shows how we work in the garden and how we select, plant and take care of the seedlings when we transplant them. For example, this is sorghum and normally we do this to ensure that the crops have enough space for growth.
03:42-03:44	FW1	What about this woman taking a bath in this dam?

03:45-4:06	R1	This woman here is showing us that when we finish working in the field and we have mud and sweat, we go and wash to clean ourselves at a dam and all this is done after we are finished with our garden work.
04:07-04:22	R1	This eighth photograph shows that after working in the garden and we have taken a bath then we travel back home to have a rest and eat. Sure.
04:23-04:40	R1	This ninth photograph shows the relish that we normally prepare. This is the easier to prepare. These are vegetables that we cook when we need to do the cooking faster. Sure.
04:41-05:01	R1	This tenth photograph shows one of the crops that we grow. This is cotton. When well-looked after, this cotton gives us more cash after sales. The money realized from the sales helps us to find food for our homes. Sure.
05:03-05:25	R1	This last photograph in this group shows the garden where we have a scheme and the engine. These are canals where we have store water to help us for our crops during the times when the water is scares.
05:26-05:37	FW1	Thank you, you really have explained nicely and clearly. Now we are done with this group. Lets now go to group two.
05:38-06:11	R1	This shows some of the crops that we have in the garden that help s when we want to just prepare our food that does not take us much time. So, this is tomato, onions that we add to our relish. And here we have maize that we do process into flour to cook our staple food. We obtain it from the market.
06:15-06:44	R1	This second photograph shows that when we are done with cooking and eating, we go to a grocery where we find things like cooking oil or soap and other items like food flavours and other additives including soda that we use in our vegetables and others. So that’s it for the second photograph.
07:13-07:24	R1	This fish is dried here and it is after it has dried that we roast or fry it.
07:25-7:39	R1	This other photograph shows the naked and partly dressed children taking time to relax as they play in the rain and others are under a tree.
07:40-07:57	R1	This other photograph also shows children playing but these ones are using toy cars under a tree and walking about to various places where they normally find as their playing area. But just by the side, there is a toilet a toilet, which is very close to the houses and it is not even protected. See there is no roof and door.
08:10-08:22	R1	This other photograph shows a woman cleaning the dishes so that when she is done with cooking she can easily use them.
08:23-09:12	R1	And this photograph here shows a carpenter and his products. Here there is one of the doors that are used when we build a house. Like in the other pictures showing houses, when we build a house it needs a door. So, it shows that a carpenter is busy working and making doors which we use on our houses for security and protection. So, that is what this man is doing here.
09:13-09:31	R1	This other photograph shows a woman who has just left the utensils from the house unattended. This is not acceptable because as you can see, there are pigs and ducks that have been attracted and are scavenging from the same utensils that will later be used by human beings. This is an agent of various diseases for us and should, at all cost, be avoided.
09:32-09:40	R1	This woman has just come from collecting firewood which is normally used in households when cooking.
09:41-10:05	R1	This photograph that children are free to play and here the child is coming back to his home after playing with friends. He is seen here playing with an old tyre whichwas abandoned and he found it useful for playing as a toy.

10:06-10:18	R1	In this photograph shows a sample of the many village houses that we have within the community. Sure.
10:23-10:43	FW1	I am seeing a woman eating Mango?
	R1	Yes, this photograph shows a woman who has just finished eating her main food, Nsima, and she feels like having something. And she is eating Mango fruit just as a supplementary food to get the maximum satisfaction.
10:44-11:11	R1	These boys and girls here, after eating, thought of coming here at this tree and relax. Some are tired and want to go and others are still playing as part of exercising.
11:13-11:25	FW1	: And this man with "Promise?"
	R1	This man with "Promise" shows that he is free at his home with his child while the wife is cooking. This shows that the man is looking after the child while the wife is cooking a sign of shared responsibility.
11:33-12:07	FW1	SO, this boys and girls, are they in Lake Malawi or Shire river?
	R1	This boys and girls are at a dam. This is the place where villagers mould bricks in this village for building houses. And during the rainy season it collect water. The water is contaminated and not good for children to swim in because all the pigs and cows do excrete in there and that is a health hazard.
12:09-15	R1	This just shows a man carrying a Bible at his home.
12:17-12:31	R1	Here it shows the family that shares its food with the neighbours. It is a common practice here to share food with those that do not have.
12:33-12:37	FW1	I am happy we have finished all in this group with a lot of photos. Thank you.
13:05-14:06	R1	We will start with this photograph, ok. This photo shows school children that have arrived early at school. And they show that they have finished doing manual work like sweeping. And as they await for a morning assembly and the beginning of classes they are captured playing as a pass time activity. They are carrying food so that they can eat and have energy whenever they are hungry.
14:08-15:00	FW1	: And the other one there?
	R1	This one here shows children coming from home after doing various house chores like sweeping around the house, cleaning utensils like pots and plates which is used for food. And here they are on their way to school for learning so that they could as well enrich themselves with academic stuff for their wellbeing. Here, in terms of food, it just shows means and ways of how people in the home do find food and when it is found how is it prepared. This is one way of teaching children how to even take care of the food before they go to school. Sure.
15:01-15:31	FW1	And down here?
	R1	This shows that these children are out for a short break after learning in class and they are relaxing. So, they decided to come here behind the school block. And when you critically look at them, you will notice that they are revising what they learnt in class. Sure.
15:38-17:44	FW1	What are they small houses for?
	R1:	These small structures are toilets and bathrooms that school children use. You may wish to know that when these children are going to school they do eat at home and is expected they may need a safe space for relieving themselves by answering nature's call and defecate in here.

	FW1:	Now you say these school-going children do eat before they go to school, does it mean they do not get food at school? The school does not provide food or for sale.
	R1:	Yes, most of these children do eat when going to school. However, there are some small-scale business persons who come from around the school to sell foodstuffs to pupils at school. They include doughnuts, banana cakes, soft drinks and others.
	R1:	You see, some of these children eat so early and by mid-day they become hungry and need to eat. So, some parents do give their children money to buy supplementary food stuffs at school like doughnuts, freezes and they eat.
	FW1:	So, they buy doughnuts and freezes. But doesn't the school provide any food?
	R1:	The only food that the school gives is education that they teach these pupils.
	FW1:	Okay, I thought there is any food provided by the school.
	R1:	No, at this school, no food is provided. It is education only that these pupils receive.

**Interview 2**		
09:40-46	R2	We will remove what we want later, we just want to arrange all the pictures first.
09:21-29	FW2	Please select those one you can explain, those ones you know you cannot explain don't take them, if there are none then there is no problem.
09:38-39	FW2	I hope you can explain all these.
09:53-54	R2	Madam, you can also select.
09:55-02	R3	Yes, I can see the pictures. This is water, at Matata.
10:08-12	FW2	Do not be forced to explain those you cannot.
10:12-13	R3	Ok.
10:15-17	R3	Lets see others.
10:26-47	FW2	We have come today to take a video, they will explain to us what is in the pictures and they will sign, we need a witness and they have picked you that's why they called you, but before I would like you to write your name and today's date. When that is done, you can leave. I don't know if you are comfortable to do that.
10:47-48	WITNES	I am.
10:48-19	FW2	Thank you. To you dad and mum, this line here is a declaration, you should confirm that MLW can take your video, photos and voice for this study. Write your name and today's date. We don't know whether it's the mum or dad who will sign.
11:23-24	R2	Please write (asking husband to sign).
11:49-55	FW2	There you should also write those words, also here as a witness you will write your name and today's date.
12:17-27	FW2	Thank you, here are your photos to be reviewed. You can kneel down and arrange them properly.
12:46-58	FW2	I would like you to explain to us the events in the pictures one by one about cooking in this household. Anyone of you can explain depending on who took the picture.
12:49-00	R2	Ok, lets do it.

13:01-03	FW2	We will start with the questions, I had forgotten.
13:01-04	R3	Ok, start with the questions.
13:06-09	R3	Can we seat down?
13:06-09	FW2	Yes, we can seat down.
13:10-20	R3	Mada! I have told you to take the kid, there goes Mada, please go to Madalitso, run (speaking to the little child).
13:20-21		May be before…
13:21-32	R3	My name is (R2). I am 28 years old, I come from Mulingama village, under the traditional authority of chief Mbendelana.
13:32-34	FW2	How many children do you have in the family?
13:34-36	R3	We have 2 children.
13:36-40	FW	Thanks you very much. We should also hear from Madam?
13:41-55	R2	My name is (R3) I am 22 years old, I come from Mulingama village, under the traditional authority of chief Mbendelana. I have 2 children, only boys.
13:55-08	FW2	In this village of Mulingama, some people are just renting while others are indigenous, what are you?
14:08-15	R2	This village is our home place.
14:15-21	FW2	How many people do you prepare food for in your house?
14:21-27	R2	I prepare food for us in the family; me, my husband and our 2 sons, 4 people in total.
14:27-34	FW2	Do the number of people you cook for change because of visitors or it remains the same always?
14:34-41	R2	If they come without a notice while we have already cooked, we just share with them what we have cooked already.
14:41-53	FW2	What are the ages of the people whom you cook food for in your house?
14:55-56	R2	Please speak (whispering to the husband).
14:53-08	R2	Most of the times the people who are around are the kids. We sometime share food with them when they are coming from school while their parents are at farms.
15:09-51	FW2	Thank you for briefing to us about your family, I now request you to explain to us the events taking place in the pictures infront of us as we don't know what they mean. Both of you can explain openly all the details for us to know as you took these pictures together. Here is a pen to act as a pointer for you. We don't know who will start.
15:53-54	R2	Ooh! Am the one to start.
15:56-57	R2	You will explain the one for fish.
15:58-00	R3	Please start with the relish one.
16:03-36	R2	This is the first picture, some of the people around here are fishermen, they usually walk around the village with the fish which we buy from them using the money we get from piece-works. Secondly, these fishermen catch and sell the fish in order to find money for their day to day lives.
16:36-47	FW2	Does this mean you cannot have any food assuming the fishermen do not come to sell you fish or when you don't have money to buy the fish?
16:48-55	R2	Food is never scarce this time due to rains. The fields have okrah which we can cook for relish.

16:55-56	FW2	OK.
16:59-30	R2	A picture about cooking nsima, This is a picture showing how to cooknsima. For us to know that food is ready, we take water and put it on fire to a moderate warmth, after that we take flour and a stirring rod. we gradually add maize flour to warm water and stir until its stiffs, if there is a lid we cover the pot.
17:30-14	R2	When the porridge is ready we take a stirring rod and flour and we keep stirring the mixture until our nsima is finally ready. When the nsima is ready we take a wooden spoon (chipande) and plates then we put them aside. We take the wooden spoon and then deep it in water so that the nsima does not stick on the wooden spoon when dishing. This is repeated until the whole nsima is dished.
18:14-34	R2	This lady is putting maize in a maize mill and this is the flour. This shows how maize flour is made out of maize grains.
18:36-40	R2	This picture, we have cooked all the food (voice cut by the husband).
18:40:42	R3	Lets start with this.
18:43-44	FW2	Please give the husband to explain.
18:44-45	R3	Ya.
18:45-46	FW2	Go on
18:47-23	R3	For this to be possible we go here. This picture shows that water is life without water man cannot live on this planet. Here we see that this lady is coming from drawing water from a borehole. Sometimes the people use unsafe water so sometimes people add chemicals like Water Guard (chlorine) to ensure that the water is safe for use. As seen from this picture, women are drawing water. The baby besides might defecate and the faeces can contaminated the water hence making it unclean.
19:23-24	FW2	Oh thanks.
19:28:30	R3	Continue!
19:32-23	R2	This is the other one. This is a market for our friends who do business for their basic needs. This picture shows a woman buying tomato and this man puts them in a plastic bag, secondly another lady went to the market to buy vegetables. For total cleanliness, she washed it and allowed it to dry in a winnower. Afterwards they allow the water to drain then cut and cook in a pot.
20:23-24	FW	Thank you.
20:24:24	R2	Yes.
20:27-43	R3	Here we see a lady cooking relish even though the lady is not shown. This picture shows that they did not protect the food from flies and germs. Had it been she cared for the food she could have covered it from flies.
20:45-51	FW2	Thank you, I assume you have covered every photo.
20:51-52	R2	Yes
20:56-11	FW2	Thank you mum and dad for your explanation from these beautiful pictures you took. We have learnt the cooking process in the villages from s
21:11-14	FW2	Thank you very much Mr. and Mrs.
21:14-15	R2 & 3	Thank you.
21:15- 17	FW2	For your participation in the study.
21:16-17	R2 &	Thank you.

Interview 3		
21:23-30	FW3	In this section, one is required to write their name and date, who is going to write?
21:31-32	R4	The father
21:39-41	FW3	Starting from the dots on top.
21:43-44	R5	Words.
21:44-45	FW3	Yes.
21:48-51	FW3	And todays date is 17th.
21:55-07	FW3	Alright family, would you please tell us about yourself, age and the number of children if you have any?
22:09-10	R5	Its ok we can explain.
22:10-11	FW3	Thank you.
22:13-42	R5	My name is (R5), aah, I am 39 years old. Aaah I have 3 children. 2 daughters and a son. This village is my home place but this is not the home place of my wife. I married her from Chapananga village.
22:42-46	FW3	How old is the wife?
22:46-01	R4	My name is (R4), I was born in 1983, I have three children; 2 daughters and 1 son. I am from Chapananga, a village in this same district of Chikhwawa.
23:01-02	FW	Thank you
23:02-02	R4	Ya
23:01-07	FW3	How many people do you cook for in this family?
23:07-16	R4	I cook for, in total we are five people; us and our three children.
23:17-23	FW3	What are the ages for all your family members?
23:23-36	R4	The first one was born in 2004, the second one in 2006, the third in 2012.
23:36-44	FW3	Are there other individuals you cook for besides your children?
23:44-48	R4	No one.
23:48-52	FW3	All these stated are the only ones you cook for?
23:52-53	R4	Yes.
23:53-54	FW3	Thank you.
23:54-57	FW3	Is there any additional information?
23:59-05	R5	Aah, There is nothing to add. You mean as pertaining to the number of people who consume food from our house?
23:06-06	FW3	Yes.
23:06:11	R5	Her explanation is ok.
24:11-17	FW3	Thank you, this time let us see the pictures that you and your wife took.
24:17-18	R5	Alright.
24:19-29	FW3	I will ask you this time to explain briefly the events taking place in the pictures, arrange them properly also and tell us in details what message i

24:29-30 R5 Alright

24:30-34 FW3 Let Us kneel down so that we can work on the pictures.

(24:34)-(28:5 Background voice, not clear (women shouting to go and draw water from the borehole).

28:55-15 FW3 You should tell us what is in the picture in relation to cooking and food and even how we get them and where they are found and what we do to find the food.

29:15-16 R4 It's farming right?

29:16-16 R5 Yes.

29:18-20 R4 Let us start with farming.

29:20-21 FW3 You want to start with the ones about farming?

29:21:21 Yes.

29:21-30 FW3 OK, you will point and explain the story in it properly for us to understand.

29:33-25 R4 In this picture, every morning we go to the farm and sometimes do piece-works if we have no money. We work at someone's field. When we get the money we go to the market to buy tomato, fish and even meat as you can see. When we come home we set fire using firewood, we chop the meat and put it on fire in a pot. We then wash the meat, put it in a pot and then boil, fry it and then add tomato and onions then finally we cook nsima.

30:25-26 FW3 Ok.

30:25-26 R4 This is a guava

30:26-28 FW3 What is happening here?

30:28-37 R4 Our children eat the guava we get from our garden.

30:37-39 FW3 As food?

30:39-40 R4 Yes.

30:40-44 FW3 Lets continue.

30:44-57 R4 But also get bananas from the fields after farming. We cut pill then boil them for breakfast for the children.

30:57-38 R5 Sometimes when one is hungry we just eat them directly from the garden, as we can see the fruits hung up. As explained already we cut bananas and make breakfast out of them, called makata and sometimes we store them until they ripe.

31:38-10 R5 This is our new type of farming because most of the times the fields where we farm here in Mlinga village are on a distance so we thought of a small garden around our home to grow vegetables for food.

32:10-32 R5 On the other hand, this is the picture of a pig. These are the animals we rear. We sometimes use them for food and money after selling

32:34-35 FW3 Has this helped you?

32:35-35 R4 A lot.

32:35-41 FW3 Do you frequently sell?

32:41-49	R4	Yes we sell rabbits then use the money to buy maize. Sometimes we sell even ducks and pigs.
32:49-51	FW3	Thank you.
32:51-52	R4	Yeah!
32:52-40	R4	Here we pluck the tree leaves of Msangu, the one over there, those ones on the fence.
32:00-01	FW3	Ok.
33:02-10	R4	We pluck and detach the leaves from the stems as seen from there
33:10-11	FW3	There?
33:11-12	R4	Yes.
33:12-27	R4	We detach and put them in a winnower then set fire and put some water until they boil. While the water is boiling, we pound some groundnuts as am doing there.
33:27-28	FW3	Where?
33:28-30	R4	Here, this is where the picture is.
33:30-31	FW	Ok.
33:31-40	R4	The water keeps boiling while we are pounding groundnuts then we add soda to the water.
33:44-49	R4	Wife asks the husband “Which one is the picture showing me pouring in a pot, was it not processed?”
33:50-12	R5	The water from Shire river was not safe. We used to fetch water direct from Shire river which was an unsafe practice that was causing a lot of diseases in our family. After they installed tap water, I can say things have improved. We have been using safe water for cooking and washing.
34:15-00	R5	This is tomato and a bin. Instead of disposing in the bin, they have carelessly disposed it, which is unclean as seen from the picture, an act that is not acceptable. There are a lot of wastes and this can lead to house flies transmitting diseases to us in our homes.
35:00-17	R5	The disposals are also close to a market, as you can see there are bananas, flies that can carry diseases to cleaner fruits like those fresh tomatoes leaving infections for the consumer because of the dust bin.
(36:17)-(36:3	Unclear Voice in background, respondent signing a declaration form, FW3 arranging papers	
**Interview 4**		
36:34-03	FW4	You may put aside those unnecessary pictures like those of just a shoe, the ones you can explain are those that were directed to you like those of food. You may now put aside those necessary pictures aside on your own.
(36:03)-(38:2	voice in background, respondent 6 & 7 arranging picture	
38:26-27	FW4	Are you ready?
38:27-28	R6 & R7	Yes.
38:28-29	FW4	Ready
38:29-29	Yes.	
38:29-40	FW4	Alright, pictures in front were taken by you but before you explain I would like to hear from you about your age starting from the husband.

38:40-43	R6	I am 34 years old.
38:43-44	FW4	You are 34?
38:44-45	R6	Yes.
38:43-48	FW4	Explain to us, how many children do you have if any?
38:48-55	R6	I have 2 children, the first one is Priscilla and the second is Hope.
38:55-57	FW4	How old is Priscilla?
38:57-01	R6	Priscilla is seven years old while Hope is two years old.
39:01-02	FW4	Hope is two years old?
39:02-03	R6	Yes.
39:03-07	FW4	Introduce yourself
39:07-44	R6	Firstly, I am (R6), a typical dweller of this village, I was born here in Chikhwawa at Mbendelana village. I am married with two children, the first born is Priscilla while the second born is Hope. Priscilla is six years old while hope is two years old. So if you would ask me to introduce myself, that's my brief account.
39:44-47	FW4	How old are you?
39:47-51	R6	I am 34 years old
39:51-54	R7	I am, firstly……..
39:54-56	FW4	Raise your voice please.
39:56-26	R7	Firstly my name is (R7). I leave in Mbendelana village. I have two children; the first one was born in 2008 on the 22nd and the other one was born in 2012 on the 5th. The first one is 6 and the last one is 2. This is my home place and I came here in 2006.
40:26-08	R6	According to the protocol given to us, we thought it wise to take photos of places where food is sold and prepared, and even where we get food. In the first place, we went into a market where they sell fish. As you can see this is a fish market where we went to take pictures of how fish is sold. As you know here in our markets here fish attracts a lot of flies which we saw could be a source of various infections to a human being. We asked the owners of how they care of the fish hygienically, stuff like that.
41:08-20	R6	They explained to us and we thought it wise of taking this picture and explain it briefly. We took about three pictures
41:21-22	FW4	This and that?
41:23-30	R6	Yes, about the market where fish is sold and how they take care of them before consumers buys, stuff like that.
41:31-38	FW4	Ok, apart from the market is there any other place where they sell fish?
41:39-52	R6	There are a lot of places, even in our locations there are smaller markets where people can sell various foods including fish.
41:53-55	FW4	What can you explain about this picture?
41:56-37	R6	This picture shows where I found a lady preparing a local drink (thobwa). We saw how this drink was brewed and taken care of before drinking. The kitchen is clean as such we thought of taking these pictures to brief them on necessary steps to be followed to prepare hygienic thobwa.
42:38-40	FW4	Was the thobwa for business or consumption?

42:0-44	R6	According to the lady she wanted to make some cash out of it.
42:45-07	R6	As you can see here this is a place where chips is prepared where I found people buying chips. The place also serves a lot of people before lunch at home or work place, so I found this man selling the chips and I had a chat with him on how he prepares the chips.
43:08-30	R6	The chips caught my attention because it was not covered, in many places the chips is put in a container for consumption while warm. The salad was also uncovered. You can see this is unprotected salad we normally consume when we want to buy chips.
43:31-53	R6	Flies can contaminate food and cause diseases so I thought it wise to take a picture and explain to the people how thing are unnecessarily done.
43:54-19	R6	This is a house where I found these kids eating. I could have given these kids a better grade (6/10) because the place was clean, the food was covered and kept in food warming containers. Stuff like that
45:19-38	R6	These are pictures of butchery where we buy meat. This is goat meat in the picture. This is in the butchery and this is the owner. What he is doing on the picture is chasing houseflies.
45:39-11	R6	So I was asking him why there were so many houseflies. I asked him if the meat was for human consumption? Then he said yes, he was selling for human consumption. As you can see from the pictures these are houseflies and we saw that they can cause several diseases to human beings so I thought it wise to chat with the seller in order to find ways of preventing spread of diseases.
45:12-12	R6	Here I found a lady making doughnuts. I found the woman busy this morning rolling doughnuts. This is the pot she used when making doughs and this is a basin wrapped by a newspaper that is touched by several people, it's also made from unhygienic materials. As per that view I saw it wasn't hygienic because the newspaper is touched by a lot of people. So people use the newspaper to roll dough-nuts and sometimes kanyenya (deep-flied meat) but the place she was using to make her dough-nuts was clean.
46:12-15	R6	This one you are seeing here is baking flour.
46:18-27	R6	This is the baking flour that she used to bake her doughnuts
46:30-43	R6	These people here were eating at a small restaurant and I found some men eating in the morning as you can see.
46:43-45	FW4	What type of food is sold at this place?
46:45-51	R6	They sell rice porridge, as seen you can see from the picture.
46:52-02	Voice from background, FW & Rs arranging pictures	
47:02-19	R6	As you can see, it's really rice porridge. I found one man who bought and was eating, I was also fortunate to meet one man who was asking to buy the same porridge.
47:19-00	R6	I just wanted to show people that it is not good to have a house that is close to a bathroom and a toilet. See, this is a toilet, it's just so close to the house and the toilets, just next is the bathroom. There are a lot of cockroaches and flies from the toilet that can carry disease-causing organism.
48:00-36	R6	So I saw that these you are seeing here are plates that she was washing just too close to the toilet. I just wanted people to see that it's not health wise to build a house close to a toilet because flies can easily transmit germs from the toilet to our food. We won't know and we will ignorantly eat the food without knowing that it is contaminated.
48:36-40	FW4	From these pictures are these charcoals, 1, 2, 3?

48:40-41	R6	Yes that's charcoal.
48:42-44	FW4	On the side of food, the topic we were discussing…. (noise of a vehicle)
48:45 – 49:22		Aerial view of Shire river…

start to end. We have learnt something.

s conveyed in each picture.
